# Sequential treatment with 5‐aza‐2′‐deoxycytidine and deacetylase inhibitors reactivates HIV‐1

**DOI:** 10.15252/emmm.201505557

**Published:** 2015-12-17

**Authors:** Sophie Bouchat, Nadège Delacourt, Anna Kula, Gilles Darcis, Benoit Van Driessche, Francis Corazza, Jean‐Stéphane Gatot, Adeline Melard, Caroline Vanhulle, Kabamba Kabeya, Marion Pardons, Véronique Avettand‐Fenoel, Nathan Clumeck, Stéphane De Wit, Olivier Rohr, Christine Rouzioux, Carine Van Lint

**Affiliations:** ^1^ Service of Molecular VirologyDepartment of Molecular Biology (DBM)Université Libre de Bruxelles (ULB)GosseliesBelgium; ^2^ Service des Maladies InfectieusesCentre Hospitalier Universitaire (CHU) de LiègeDomaine Universitaire du Sart‐TilmanUniversité de LiègeLiègeBelgium; ^3^ Laboratory of ImmunologyIRISLabCHU‐BrugmannUniversité Libre de Bruxelles (ULB)BrusselsBelgium; ^4^ Service de VirologieEA7327AP‐HPHôpital Necker‐Enfants‐MaladesUniversité Paris‐DescartesParisFrance; ^5^ Service des Maladies InfectieusesCHU St‐PierreUniversité Libre de Bruxelles (ULB)BrusselsBelgium; ^6^ IUT Louis Pasteur de SchiltigheimUniversity of StrasbourgSchiltigheimFrance; ^7^ Institut Universitaire de France (IUF)ParisFrance; ^8^ Service de GénétiqueCentre Hospitalier Universitaire (CHU) de LiègeDomaine Universitaire du Sart‐TilmanLiègeBelgium

**Keywords:** demethylating agent, epigenetics, HDACIs, HIV latency, HIV reservoir, Microbiology, Virology & Host Pathogen Interaction, Pharmacology & Drug Discovery

## Abstract

Reactivation of HIV gene expression in latently infected cells together with an efficient cART has been proposed as an adjuvant therapy aimed at eliminating/decreasing the reservoir size. Results from HIV clinical trials using deacetylase inhibitors (HDACIs) question the efficiency of these latency‐reversing agents (LRAs) used alone and underline the need to evaluate other LRAs in combination with HDACIs. Here, we evaluated the therapeutic potential of a demethylating agent (5‐AzadC) in combination with clinically tolerable HDACIs in reactivating HIV‐1 from latency first *in vitro* and next *ex vivo*. We showed that a sequential treatment with 5‐AzadC and HDACIs was more effective than the corresponding simultaneous treatment both *in vitro* and *ex vivo*. Interestingly, only two of the sequential LRA combinatory treatments tested induced HIV‐1 particle recovery in a higher manner than the drugs alone *ex vivo* and at concentrations lower than the human tolerable plasmatic concentrations. Taken together, our data reveal the benefit of using combinations of 5‐AzadC with an HDACI and, for the first time, the importance of treatment time schedule for LRA combinations in order to reactivate HIV.

## Introduction

Thirty years after its discovery, human immunodeficiency virus type 1 (HIV‐1) remains a major problem of public health. Combination antiretroviral therapy (cART) is potent but not curative. cART requires lifelong adherence and does not fully restore health or a normal immune status in HIV‐1‐infected individuals. Although multiple reservoirs may exist, the HIV‐1 reservoirs containing stably integrated, transcriptionally silent but replication‐competent proviruses are recognized to predominate among infected CD4^+^ T cells (Eisele & Siliciano, 2012). They are therefore a permanent source for virus reactivation and could be responsible for the rebound of plasma viremia observed after cART interruption (Tyagi & Bukrinsky, 2012). Persistence of truly latent (i.e. non‐defective) HIV‐1 proviruses represents a major obstacle to eradication, as suggested by the failure of cART intensification strategies at clearing the viral reservoirs (Dinoso *et al*, 2009; Gandhi *et al*, 2010; Yukl *et al*, 2010). Indeed, the levels of HIV‐1 reservoirs appear as one of the critical factors influencing the duration of a remission after cART cessation (Saez‐Cirion *et al*, 2013). Consequently, a decline of the HIV‐1 latent reservoirs to a level sufficient to permit an efficient control of the infection by the host immune system might allow interruptions in therapy (“treatment‐free windows”). Reactivation of HIV gene expression in latently infected cells together with an efficient or intensified cART could serve as an adjuvant therapy aimed at eliminating/decreasing the pool of latent viral reservoirs.

The chromatin organization and the epigenetic control of the HIV‐1 promoter are key elements in transcriptional silencing (Van Lint *et al*, 2013). The repressive nucleosome nuc‐1, located immediately downstream of the transcription start site, is maintained hypoacetylated by histone deacetylases (HDACs) in latent conditions (Verdin *et al*, 1993; Van Lint *et al*, 1996). The use of HDAC inhibitors (HDACIs) as latency‐reversing agents (LRAs) has been well characterized in several latency models and in *ex vivo* cART‐treated HIV‐1^+^ patient cell cultures (Quivy *et al*, 2002; Archin *et al*, 2009; Contreras *et al*, 2009; Reuse *et al*, 2009; Matalon *et al*, 2010). Several anti‐HIV latency clinical studies and trials using HDACIs have been reported in the HIV field [VPA (Lehrman *et al*, 2005; Siliciano *et al*, 2007; Archin *et al*, 2008, 2010; Sagot‐Lerolle *et al*, 2008; Routy *et al*, 2012a,b), SAHA (Archin *et al*, 2012, 2014; Elliott *et al*, 2014), panobinostat (Rasmussen *et al*, 2015), and romidepsin (Sogaard *et al*, 2015)]. Altogether, these studies are encouraging but question the efficiency of HDACIs used alone to reduce the size of the HIV‐1 reservoirs and underline the need to evaluate other classes of LRAs, alone or in combination with HDACIs.

Targeting simultaneously different mechanisms of latency should be more efficient when viral eradication/remission is the objective since the combination of different classes of compounds could synergize (i.e. result in a higher reactivation level than the sum of the reactivations produced by each compound individually) to reactivate HIV expression in latently infected cells. In this regard, we have previously demonstrated proof‐of‐concepts for the coadministration of two different classes of promising LRAs [an NF‐κB inducer + an HDACI (Quivy *et al*, 2002; Adam *et al*, 2003; Reuse *et al*, 2009), an NF‐κB inducer + a P‐TEFb‐releasing agent (Darcis *et al*, 2015), a histone methyltransferase inhibitor (HMTI) + an HDACI (Bouchat *et al*, 2012), an HMTI + an NF‐κB inducer (Bouchat *et al*, 2012)] as a therapeutic perspective to decrease the pool of latent HIV‐1 reservoirs in the presence of efficient cART. Epigenetically, it is known that DNA methylation and histone deacetylation cooperate to establish and maintain a heterochromatin environment. In the case of HIV, the HIV‐1 promoter has been previously shown to be hypermethylated *ex vivo* and resistant to reactivation in the latent reservoirs from cART‐treated aviremic HIV‐1 infected individuals, as opposed to the hypomethylated 5′ LTR of integrated proviruses present in viremic patients (Blazkova *et al*, 2009). Although controversy remains about the level of DNA methylation in patient cells *in vivo* (Blazkova *et al*, 2009, 2012; Palacios *et al*, 2012; Ho *et al*, 2013), the DNA methylation status of the HIV‐1 promoter could contribute to “lock” the silent state of the provirus in cooperation with histone repressive post‐translational modifications such as histone deacetylation, thereby making the return of the provirus to an active state more difficult (Blazkova *et al*, 2009). In this view, demethylating agents could represent promising candidate drugs in combination with HDACIs for reducing the pool of latent HIV reservoirs. Two well‐characterized nucleoside analog DNA methylation inhibitors, 5‐azacytidine (5‐AzaC, marketed as Vidaza) and 5‐aza‐2′‐deoxycytidine (5‐AzadC, marketed as Dacogen), are currently FDA‐approved to treat myelodysplastic syndrome and used in cancer therapies (Kantarjian *et al*, 2006). Few studies have already tested the HIV‐1 reactivation potential of 5‐AzadC + HDACI combinatory treatments using latently infected cell lines but have failed to show any synergistic effect *in vitro* (Blazkova *et al*, 2009; Kauder *et al*, 2009; Fernandez & Zeichner, 2010).

In this report, we thoroughly studied the sequential aspect of cellular treatments combining demethylating agents with clinically tolerable HDACIs in latently infected T‐cell lines and in *ex vivo* cultures of CD8^+^‐depleted PBMCs or resting CD4^+^ T cells from cART‐treated aviremic HIV‐1^+^ patients. We demonstrated that these two classes of LRAs synergistically reactivated HIV in the context of sequential treatments. Moreover, we determined their metabolic activity profiles and their impact on global T‐cell activation. Taken together, our data reveal the benefit of using combinations of a demethylating agent and an HDACI and, for the first time, the importance of treatment time schedule for LRA combinations in order to reactivate HIV.

## Results

### The DNA methylation inhibitor 5‐AzadC induces HIV‐1 transcription and production in a latently infected T‐cell line

Several postintegration latency models exist to study the mechanisms of transcriptional reactivation and the pathogenesis of HIV‐1. In order to test the HIV‐1 reactivation potential of 5‐AzaC and 5‐AzadC DNA methylation inhibitors, we used the HIV‐1 latently infected J‐Lat 8.4 cell line since the Verdin's laboratory has previously reported that two CpG islands flanking the transcription start site are hypermethylated in several latently infected J‐Lat cell lines (Kauder *et al*, 2009).

**Figure 1:**
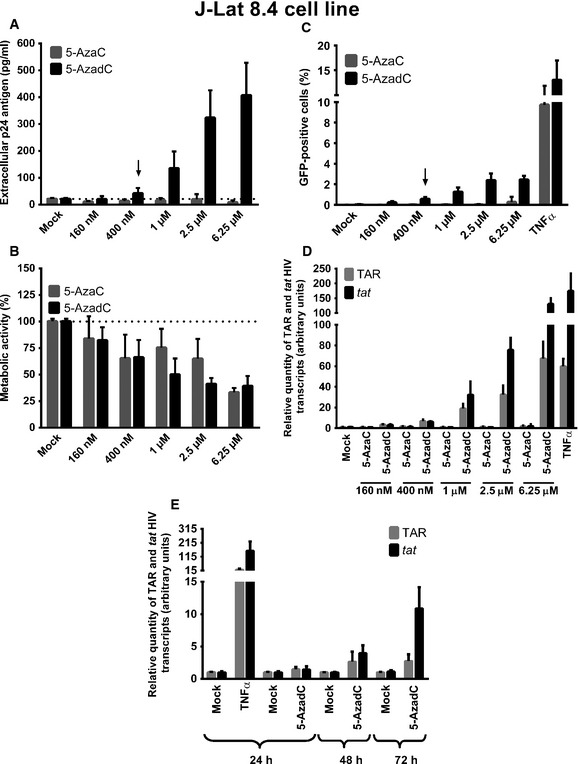
The DNA methylation inhibitor 5‐AzadC induces HIV‐1 expression in latently infected T cells A–DJ‐Lat 8.4 cells were mock‐treated or treated with increasing concentrations of 5‐AzadC or 5‐AzaC. At 72 h post‐treatment, viral production was measured by quantifying p24 antigen production in culture supernatants (A); metabolic activity was assessed by a WST‐1 assay (B); viral protein expression was analyzed by FACS (C); and initiated (primers TAR) or elongated (primers *tat*) transcripts were quantified by RT–qPCR (D). The selected dose was indicated by an arrow.EJ‐Lat 8.4 cells were mock‐treated or treated with 5‐AzadC (400 nM) or TNF‐α (10 ng/ml) as a positive control. At 24, 48 or 72 h post‐treatment, initiated (primers TAR) or elongated (primers *tat*) transcripts were quantified by RT–qPCR.Data information: For (D, E), results were normalized using β‐actin gene primers and are presented as histograms indicating the fold inductions compared to mock‐treated condition for each time period. For (A–E), means and standard errors of the means from three independent biological duplicates (*n *=* *6) are indicated. The result obtained with mock‐treated cells was arbitrarily set at a value of 1 (D, E) and 100% (B).

As these drugs are nucleoside analogs and are incorporated into DNA or RNA, we performed stimulation kinetics (24, 48 and 72 h) and only obtained viral production in culture supernatants after 72 h of treatment. Therefore, Fig 1A shows only the data for the 72‐h time point. At 72 h post‐treatment, we observed that 5‐AzadC, but not 5‐AzaC at the same doses, induced viral production in a dose‐dependent manner from 400 nM to 6.25 μM (Fig 1A). WST‐1 assays revealed that metabolic activities decreased in a dose‐dependent manner from 400 nM ranging from 66.3% to 39.3% when using increasing 5‐AzadC concentrations (Fig 1B). We confirmed the potency of 5‐AzadC in comparison with 5‐AzaC (observed by quantification of p24 viral production in Fig 1A) by showing that 5‐AzadC increased the number of GFP‐positive cells (assessed by FACS in Fig 1C) and increased viral mRNA expression (assessed by RT–qPCR in Fig 1D). Of note, relative levels of initiated (TAR) transcripts were lower than those of elongated (*tat)* transcripts for all conditions as compared to mock‐treated condition. This phenomenon can be explained by the fact that more TAR transcripts are detected in mock‐treated condition due to RNA polymerase II pausing present in latency condition. We also analyzed the mean fluorescence intensities (MFI) of the GFP‐positive cell populations following increasing concentrations of 5‐AzadC (Appendix Fig S1), and we showed that the amount of GFP produced per cell was also increased, indicating an enhanced HIV‐1 gene expression.

As 5‐AzadC reactivated HIV‐1 from latency at a concentration of 400 nM (Fig 1A) that is lower than the tolerable peak of plasmatic concentration (*C*_max_) after usual dosage (20 mg/m²) in human anticancer therapy [around 650 nM (Inc E (2014) Dacogen (decitabine) for injection, full prescribing information)], we decided to use 400 nM of 5‐AzadC as working concentration in our next experiments (indicated by an arrow, Fig 1A). Study of the 5‐AzaC reactivation potential at higher doses than the ones we used had no interest because the *C*_max_ after usual dosage (75 mg/m²) in human anticancer therapy is around 3 μM for 5‐AzaC (Laille *et al*, 2014). The higher reactivation potential of 5‐AzadC compared to 5‐AzaC can be explained by the different intracellular metabolisms of these two drugs (Li *et al*, 1970). Indeed, in contrast to its reduced analog 5‐AzadC, 5‐AzaC is a ribonucleoside and has to be first reduced in a deoxynucleoside via a limiting enzymatic step before being incorporated into DNA. Moreover, while 5‐AzadC incorporates exclusively into DNA, only a small percentage (10–20%) of 5‐AzaC is incorporated into DNA, the remainder being incorporated into RNA (Li *et al*, 1970).

Since a 72‐h treatment with 5‐AzadC was required to observe an increase in HIV‐1 expression (see here above), we performed kinetics studies to follow the viral mRNA level increase in response to 5‐AzadC treatment. As shown in Fig 1E, 5‐AzadC caused an increased expression of both initiated (TAR) and elongated (*tat*) HIV‐1 transcripts, which was the highest at 72 h post‐treatment, whereas a 24‐h treatment was sufficient to observe the effect of the NF‐κB inducer TNF‐α on viral transcriptional activity (Fig 1E).

In conclusion, we demonstrated for the first time that 5‐AzadC, in contrast to 5‐AzaC, reactivated HIV‐1 expression from latency in latently infected J‐Lat 8.4 cells treated for 72 h, time needed to obtain an effective removal of the viral transcription block, probably due, at least partially, to DNA demethylation as previously shown by Verdin and colleagues (Kauder *et al*, 2009). Consequently, Dacogen (5‐AzadC), but not Vidaza (5‐AzaC), used at concentrations lower than that generally achieved in human cancers could be a promising LRA and be used in combination with other LRAs in reactivation strategies aimed at reducing the HIV‐1 reservoirs.

### Determination of an optimal concentration of several clinically tolerable HDACIs used in human therapy to induce HIV‐1 production in a latently infected T‐cell line

In order to highlight new therapeutic approaches to purge latent HIV reservoirs, we selected some clinically tolerable HDACIs that could be administrated in future HIV clinical trials. We compared the reactivation potentials of HDACIs previously extensively tested in several HIV‐1 reactivation studies (VPA, NaBut, MS‐275, and SAHA) (reviewed in Van Lint *et al*, 2013) to those of three promising and more recently tested HDACIs (belinostat, panobinostat, and romidepsin) (Rasmussen *et al*, 2013; Wei *et al*, 2014). Table 1 describes the characteristics of these HDACIs.

**Table 1:** Characteristics of used HDACIs

Acronym in this study	Name	Marketed as	Approved for	Treatment corresponding to *C*_max_	Human tolerable plasmatic concentration (*C*_max_)	Selected doses in this study	Clinical trials in HIV field
MS‐275	Entinostat	n.a.	Ongoing clinical trials for the treatment of various cancers	For an usual dosage	0.14 ± 0.24–0.3 ± 0.22 μM (Wightman *et al*, 2013)	0.5 μM	n.a.
NaBut	Sodium butyrate	Buphenyl	Sickle cell anemia and beta‐thalassemia	For 27 and 36 g/day	1.225 and 1.605 mM (Phuphanich *et al*, 2005)	1.5 mM	n.a.
SAHA	Vorinostat, suberoylanilide hydroxamic acid	Zolinza	Cutaneous T‐cell lymphoma	For an usual dosage two times/day during 2–3 weeks	0.3–1.7 μM (Merck (2013) Zolinza (vorinostat) prescribing information)	1.25 μM	Archin *et al* (2014, 2012), Elliott *et al* (2014)
VPA	Valproic acid	Depakine	Chronic neurological and psychiatric disorders	For an usual dosage	0.25–0.5 mM (AbbVie (2014) Depakote prescribing information)	2.5 mM	Archin *et al* (2010, 2008), Lehrman *et al* (2005), Routy *et al* (2012a,b), Sagot‐Lerolle *et al* (2008), Siliciano *et al* (2007)
Beli	Belinostat, PXD101	Beleodaq	Relapsed or refractory peripheral T‐cell lymphoma	1,000 mg/m² for five consecutive days	> 1 μM (Steele *et al*, 2011)	1 μM	n.a.
Pano	Panobinostat, LBH‐589	Faridak	Myeloma therapy	For one dose with an usual dosage	0.6–1.4 μM (Rathkopf *et al*, 2010)	0.15 μM	Rasmussen *et al* (2015)
Romi	Romidepsin, FK228	Istodax	Peripheral T‐cell lymphoma or cutaneous T‐cell lymphoma	14 mg/m²	0.112 μM (Celgene (2014) Istodax prescribing information)	0.0175 μM	Sogaard *et al* (2015)

**Figure 2:**
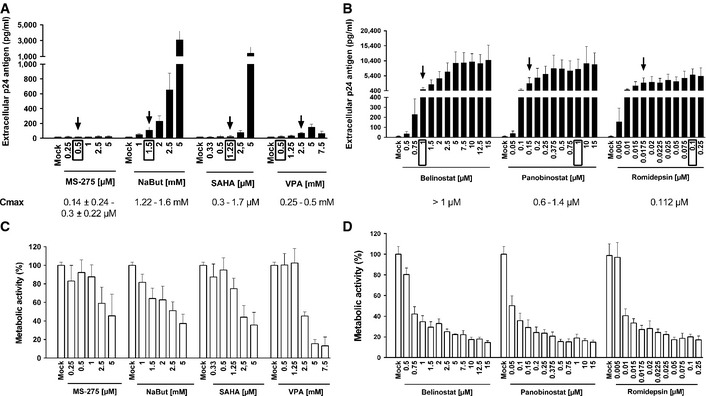
Determination of an optimal concentration for each HDACI to induce HIV‐1 production in latently infected cells A–DJ‐Lat 8.4 cells were mock‐treated or treated with increasing concentrations of HDACIs. At 24 h post‐treatment, viral production was measured by quantifying p24 antigen production in culture supernatants (A, B) and metabolic activity was assessed by a WST‐1 assay (C, D). Means and standard errors of the means from three independent biological duplicates (*n *=* *6) are indicated. The result obtained with mock‐treated cells was arbitrarily set at a value of 100% (C, D). The selected doses are indicated by an arrow. Plasmatic concentrations after usual dosage (*C*_max_) of each drug in human therapy are indicated below the LRA names and by a box in the graph.

As shown in Fig 2, all selected HDACIs, except MS‐275, induced viral production after 24 h in a dose‐dependent manner in the latently infected J‐Lat 8.4 cell line (Fig 2A and B). This measurement is commonly performed 24 h post‐treatment for HDACIs in *in vitro* HIV reactivation experiments (Reuse *et al*, 2009). The absence of reactivation with MS‐275 was in agreement with results previously reported (Reuse *et al*, 2009; Wightman *et al*, 2013). Of note, in the Lewin's study (Wightman *et al*, 2013), a significant viral production has been detected following MS‐275 treatment after 48 h of stimulation in the ACH2 cell line and after 3 days of stimulation in a primary CD4^+^ T‐cell model for HIV‐1 latency.

As shown in Fig 2A, NaBut presented a higher reactivation potential than those observed with VPA, MS‐275, and SAHA. However, the HDACIs belinostat, panobinostat, and romidepsin induced higher HIV‐1 production than VPA, NaBut, MS‐275, and SAHA (Fig 2B, compared to Fig 2A). Moreover, if we only considered the HDACI concentrations close to their *C*_max_ (indicated by a box in Fig 2A and B), panobinostat and romidepsin were more potent than the other HDACIs (including belinostat). Taken together, we confirmed and extended previous works (Rasmussen *et al*, 2013; Wei *et al*, 2014) by comparing the HIV‐1 reactivation potentials of several HDACIs. This allowed us to determine for each HDACI an optimal concentration (outlined by an arrow in Fig 2A and B) in terms of both their HIV‐1 reactivation potential and their *C*_max_ (mentioned at the bottom of Fig 2A and B). In our next experiments, we selected the following HDACI concentrations for combinatory treatments with 5‐AzadC: (i) MS‐275 at a concentration of 0.5 μM in agreement with *C*_max_; (ii) NaBut at 1.5 mM consistent with *C*_max_; (iii) SAHA at 1.25 μM in agreement with *C*_max_; (iv) VPA at 2.5 mM, which is higher than *C*_max_ but allowed HIV reactivation as opposed to VPA reactivation incapacity at *C*_max_ used in clinical trials (Lehrman *et al*, 2005; Siliciano *et al*, 2007; Archin *et al*, 2008, 2010; Sagot‐Lerolle *et al*, 2008; Routy *et al*, 2012a,b); (v) belinostat at 1 μM consistent with *C*_max_; (vi) panobinostat at 0.15 μM corresponding to the dose initiating the plateau phase; and (vii) romidepsin at 0.0175 μM corresponding to an intermediate dose between the concentration initiating the plateau phase and *C*_max_.

In parallel, we assessed cellular metabolic activities after treatment with increasing HDACI concentrations and observed dose‐dependent decreases (Fig 2C and D). Those decreases were drastic for belinostat, panobinostat, and romidepsin after the second tested dose despite their approval in human therapy (Fig 2D). Metabolic activity decrease did not seem to be due to viral cytopathic effects but only to intrinsic toxicity of the drugs since the same profiles were observed after LRA treatment of the uninfected Jurkat cell line [the parental cell line of the J‐Lat cell clones (Jordan *et al*, 2003)] (Appendix Fig S2).

In conclusion, we determined for each HDACI an optimal concentration based on its HIV‐1 reactivation potential in a latently infected T‐cell line and on its *C*_max_. We demonstrated that panobinostat and romidepsin were more potent than the other HDACIs tested at a dose 4‐ to 9.3‐fold and 6.4‐fold inferior to their corresponding *C*_max_, respectively.

### Sequential treatment with the DNA methylation inhibitor 5‐AzadC and several HDACIs synergistically activates HIV‐1 gene expression and production in latently infected T‐cell lines

DNA methylation and histone deacetylation contribute to gene silencing. Consequently, both mechanisms could also cooperate to establish a heterochromatin environment.

**Figure 3:**
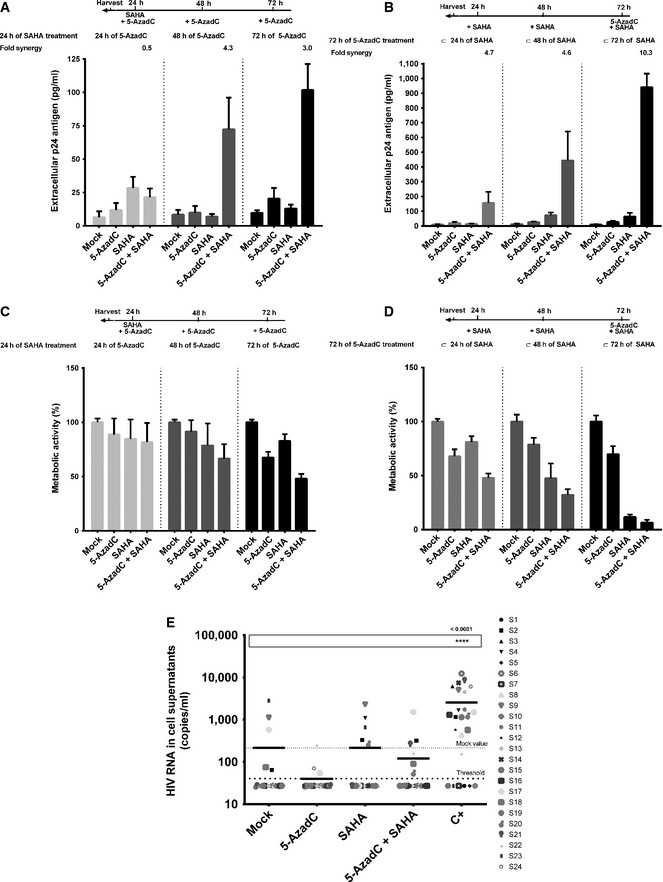
Determination of 5‐AzadC + SAHA treatment schedule *in vitro* and *ex vivo* A–DJ‐Lat 8.4 cell line was mock‐treated or treated with 5‐AzadC and/or SAHA for different periods of time as indicated. Samples were harvested at the indicated times. Viral production was measured by quantifying p24 antigen production in culture supernatants (A, B) and metabolic activity was assessed by a WST‐1 assay (C, D). Means and standard errors of the means from three independent biological duplicates (*n *=* *6) are indicated. The result obtained with mock‐treated cells was arbitrarily set at a value of 100% (C, D).EFrom data of *ex vivo* cultures of CD8^+^‐depleted PBMCs isolated from 24 HIV ^+^ patients presented in Appendix Table S2, the extracellular HIV‐1 genomic RNA levels for each LRA treatment are represented. One night after cell purification, cells were mock‐treated or simultaneously treated with 5‐AzadC (1 μM) and/or SAHA (1 μM). Six days after treatment, the concentration of viral RNA in culture supernatants was determined (in copies/ml). The results were reported as the actual HIV RNA copy numbers/ml or as an estimated value calculated as 50% of the smallest value when HIV RNA was not detected in order to assign a log value. Means are represented. Nonparametric one‐way ANOVA for independent samples (Kruskal–Wallis) followed by paired comparisons between each treated condition and the mock‐treated condition (Mann–Whitney test) are performed.

To evaluate the reactivation potential of treatments combining an HDACI and 5‐AzadC in latently infected J‐Lat 8.4 cells, we measured HIV‐1 production after sequential or simultaneous treatment in order to determine whether 5‐AzadC treatment could have a favorable effect on viral reactivation induced by HDACIs. To this end, we progressively increased the time of 5‐AzadC treatment (24, 48 and 72 h) while maintaining a constant 24‐h SAHA treatment before harvesting the cells. More precisely, treatments were as follows: (i) a simultaneous 24‐h treatment with both 5‐AzadC and SAHA; (ii) a 24‐h 5‐AzadC pretreatment followed by a 24‐h SAHA induction, corresponding to a total 48‐h 5‐AzadC treatment; and (iii) a 48‐h 5‐AzadC pretreatment followed by a 24‐h SAHA induction, corresponding to a total 72‐h 5‐AzadC treatment. By extracellular p24 ELISA assays, we observed that 5‐AzadC synergistically increased the SAHA reactivation potential after 48 h and 72 h of 5‐AzadC treatment (Fig 3A and Appendix Table S1). Of note, the fold synergy was calculated by dividing the effect observed after the LRA combined treatment by the sum of the effects obtained after the individual LRA treatments (Herschlag & Johnson, 1993). For instance, for 72 h of 5‐AzadC treatment including 24 h of SAHA treatment, we have calculated the fold synergy in the following manner: 5‐AzadC treatment led to a 2.09‐fold induction of viral production in J‐Lat 8.4, SAHA treatment led to a 1.33‐fold induction, and 5‐AzadC + SAHA treatment led to a 10.41‐fold induction (Appendix Table S1). This amount of viral production is 3.04‐fold greater than the sum of the effects produced by each activator separately [3.04‐fold synergism = 10.41/(2.09 + 1.33)]. This method allows us to determine whether our effect was synergic or not when the fold synergy is above 1 as described in Herschlag and Johnson (1993). The fold synergy data were presented at the top of the histograms presenting p24 levels. As shown in Fig 3A, sequential treatments resulted in highest synergistic activations after 48‐h 5‐AzadC treatment and with the maximal HIV production being reached after 72 h of 5‐AzadC treatment. These data highlighted for the first time the importance of treatment time schedule for LRA combinations. The setup of LRA concentrations but also the sequential aspect of the combined treatments was thus critical to reach synergistic activation of HIV‐1 production since previous reports testing 5‐AzadC in combination with HDACIs had failed to show any synergistic effect (Blazkova *et al*, 2009; Kauder *et al*, 2009; Fernandez & Zeichner, 2010). As shown in Fig 3B, we observed that the progressively increased times of SAHA treatment (24, 48 and 72 h) with a constant 72‐h 5‐AzadC treatment were associated with higher HIV productions than those observed in Fig 3A, with a maximal HIV production for the simultaneous 72‐h treatment. However, due to a metabolic activity drastically altered in these conditions compared to a constant 24‐h SAHA treatment (Fig 3D compared to Fig 3C), this simultaneous 72‐h treatment could not be used in our next experiments. Importantly, in *ex vivo* cultures of CD8^+^‐depleted PBMCs from 24 aviremic cART‐treated HIV^+^ patients, we observed that the simultaneous treatment with 5‐AzadC + SAHA weakly increased the percentage of reactivated patient cell cultures (Appendix Table S2), but did not cause a higher HIV recovery than that obtained in the mock‐treated condition (Fig 3E). Of note, in this latter experiment, the positive control did cause a statistically relevant increase HIV recovery compared to the mock condition. Consequently, in our next experiments, we used a sequential time schedule where J‐Lat 8.4 and 15.4 cells were first mock‐treated or treated with 5‐AzadC for 48 h and then mock‐treated or treated with HDACIs for 24 h. After this 72‐h sequential treatment, we analyzed HIV‐1 gene expression.

**Figure 4:**
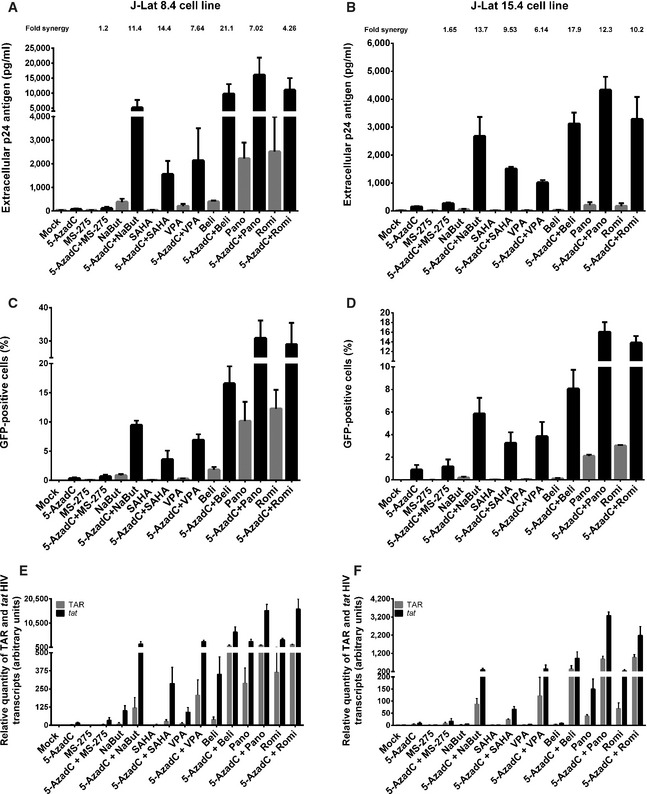
Sequential 5‐AzadC + HDACI treatments synergistically activate HIV‐1 gene expression and production in latently infected cells A–FJ‐Lat 8.4 (A, C, E) and 15.4 (B, D, F) cell lines were mock‐treated or treated with 5‐AzadC. At 48 h post‐treatment, HDACIs were then added for 24 h. At 72 h 5‐AzadC post‐treatment, samples were harvested and analyzed as follows: viral p24 production in the cell supernatant was measured (A, B); FACS analyses were performed and the percentages of GFP ^+^ cells are presented as histograms (C, D); initiated (primers TAR) or elongated (primers *tat*) transcripts were quantified by RT–qPCR, and results were normalized using the β‐actin gene primers and are presented as histograms representing fold inductions compared to the mock‐treated condition (E, F). Means and standard errors of the means from three independent biological duplicates (*n *=* *6) are indicated. The result obtained with mock‐treated cells was arbitrarily set at a value of 1 (E, F).

As shown in Fig 4A, individual treatments with 5‐AzadC or HDACIs activated HIV‐1 production in the J‐Lat 8.4 cell line. Remarkably, when cells were treated with both drugs, we observed important synergistic inductions of viral production, except for the 5‐AzadC + MS‐275 treatment (Fig 4A and Appendix Table S3). Treatments with 5‐AzadC + belinostat, 5‐AzadC + panobinostat, and 5‐AzadC + romidepsin exhibited the highest viral productions, and the 5‐AzadC + belinostat combination presented the highest fold synergy (Fig 4A and Appendix Table S3). Similar results were obtained in the J‐Lat 15.4 cell line (Fig 4B and Appendix Table S3).

Importantly, we demonstrated using J‐Lat cell lines that the synergistic effects were not only due to an enhanced HIV‐1 expression from cells whose transcription was already reactivated by the drugs used alone but also due to the recruitment of unresponsive cells into the responding population as assessed by FACS analysis (Fig 4C and D). Combinatory treatments 5‐AzadC + romidepsin and 5‐AzadC + panobinostat induced HIV‐1 expression in a higher proportion of cells than the drugs alone and than the other combinations we tested, with percentages of J‐Lat 8.4 GFP‐positive cells of 28.9 and 30.8% and of J‐Lat 15.4 GFP‐positive cells of 13.8 and 16.0%, respectively (Fig 4C and D). We also analyzed the mean fluorescence intensities (MFI) of the GFP‐positive cell populations following the 5‐AzadC + HDACIs treatments (Appendix Fig S3A and B), and we showed that the amount of GFP produced per cell was also more potently increased as compared to individual LRA treatments. These data showed that synergy was due to both an increase in the number of cells expressing virus and an enhanced HIV‐1 gene expression.

In order to test the effect of combined 5‐AzadC + HDACIs treatments on HIV‐1 promoter transcriptional activity, initiated (TAR) versus elongated (*tat*) HIV‐1 transcripts were measured by RT–qPCRs. As shown in Fig 4E, treatments with 5‐AzadC alone or with HDACIs alone (except for panobinostat and romidepsin) increased the relative amount of both initiated and elongated viral transcripts. Importantly, 5‐AzadC + HDACIs combined treatments (especially 5‐AzadC + panobinostat and 5‐AzadC + romidepsin) caused higher accumulations of initiated and elongated transcripts than those caused by the drugs alone and by the other combinations tested (Fig 4E). Similar results were obtained in the J‐Lat 15.4 cell line (Fig 4F).

In conclusion, sequentially combined 5‐AzadC + HDACIs treatments synergistically activated HIV‐1 transcription and production in two latently infected T‐cell lines. Moreover, these combinations removed the block to viral transcription observed in latently infected J‐Lat cells in a superior manner than the drugs alone. These combinations also induced HIV‐1 expression in a higher proportion of cells than the drugs alone. Altogether, our *in vitro* results in latently infected cell lines suggested that the 5‐AzadC + panobinostat and 5‐AzadC + romidepsin combinations were promising in order to reactivate HIV and prompted us to test these types of combinations in *ex vivo* cultures of patient cells.

### HDACIs alone or in combination with 5‐AzadC induce a variation in cellular metabolic activities depending on the treatment in CD8^+^‐depleted PBMCs from HIV‐negative donors

**Figure 5:**
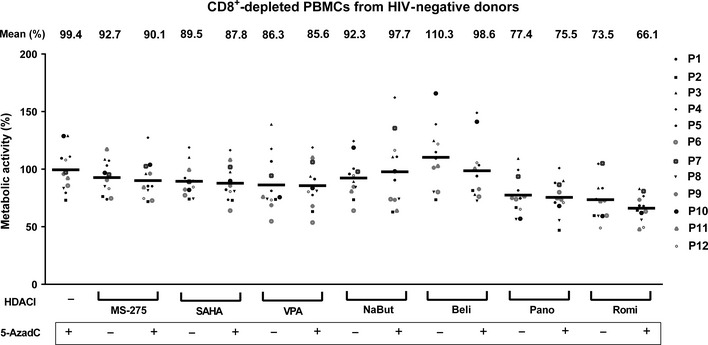
Sequential 5‐AzadC + HDACI treatments induced a variation of cellular metabolic activity depending on the treatment in CD8^+^‐depleted PBMCs from HIV‐negative donors One night after cell purification, CD8^+^‐depleted PBMCs from 12 HIV‐negative donors were mock‐treated or treated with 5‐AzadC. Three days post‐treatment, 1/3 of medium was replaced and HDACIs were added to the cultures. Six days after 5‐AzadC treatment, WST‐1 assay was performed. Mock value was arbitrarily fixed at 100% for each individual. Means are indicated by a line.

We next evaluated whether the combined treatments had an effect on metabolic activity of uninfected CD8^+^‐depleted PBMCs. To this end, we prepared *ex vivo* cultures of CD8^+^‐depleted PBMCs from 12 HIV‐negative donors and we stimulated these cultures with 5‐AzadC and HDACIs used sequentially. In order to transpose the sequential time schedule used in cell lines to primary cells, cells were mock‐treated or treated with 5‐AzadC one night after cell purification from blood. Three days after the 5‐AzadC treatment, HDACIs or control medium was added to the cell cultures. Six days after 5‐AzadC treatment, WST‐1 assays were performed (Fig 5). Mock‐treated condition value was arbitrarily set at 100% for each donor. As shown in Fig 5, treatment with 5‐AzadC did not affect the mean of cellular metabolic activities obtained from the 12 HIV‐negative donors. Mean metabolic activities were slightly altered after HDACI treatments (alterations of 7.3–26.5%) in contrast to the important decreases in metabolic activities we observed in the latently infected J‐Lat 8.4 and uninfected Jurkat cell lines (compare Fig 5 with Appendix Fig S2). This discrepancy could be explained by the fact that HDACIs induce a cancer cell‐specific cytotoxicity. Indeed, a previous study has shown that panobinostat presents toxicity in transformed cell lines but is relatively sparring in primary cells (Prince *et al*, 2009). Importantly, we observed no additional decrease in the mean of metabolic activities following the combined 5‐AzadC + HDACI treatments as compared to individual treatments, except for the 5‐AzadC + romidepsin treatment for which we observed a decrease in metabolic activity as compared to individual romidepsin treatment (66.1% compared to 73.5%). In conclusion, we highlighted that 5‐AzadC alone and all 5‐AzadC + HDACI treatments induced weak decreases in mean metabolic activities (up to a 33.9% maximal decrease) depending on the treatment as compared to the mean metabolic activities observed in mock condition.

### 5‐AzadC + panobinostat and 5‐AzadC + romidepsin sequential treatments induce HIV recovery in CD8^+^‐depleted PBMCs isolated from cART‐treated aviremic HIV^+^ patients

To address the physiological relevance of our *in vitro* reactivation results, we investigated the reactivation potential of the 5‐AzadC + HDACI combinations in *ex vivo* cultures of cells isolated from cART‐treated aviremic HIV‐1^+^ patients. We purified CD8^+^‐depleted PBMCs from the blood of 19 selected volunteer patients (Table 2). In order to evaluate the frequency of infected cells during plating, we quantified cell‐associated total HIV‐1 DNA. One night after purification, cells were either mock‐treated or treated with 5‐AzadC and/or HDACIs (according to the sequential time schedule described above) or with anti‐CD3 + anti‐CD28 antibodies as positive control (Pierres *et al*, 1988; Costello *et al*, 1993). Importantly, in all our experiments, purified cells were cultured in the absence of both IL‐2 and allogenic stimulation in order to avoid non‐specific global T‐cell activation and proliferation, which might cause an increase in genomic viral RNA level. Six days after treatment, we measured HIV‐1 genomic RNA concentrations in culture supernatants.

**Table 2:**
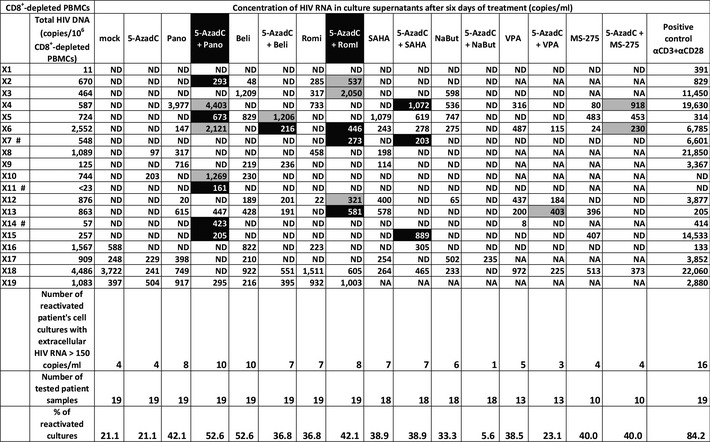
Representation of reactivation status of *ex vivo* cultures of CD8^+^‐depleted PBMCs isolated from HIV^+^ patients

One night after cell purification, cells were mock‐treated or treated with 5‐AzadC. Three days post‐treatment, 1/3 of medium was removed and HDACIs were added in the cultures. Six days after 5‐AzadC treatment, the concentration of viral RNA in culture supernatants was determined (in copies/ml; ND means undetectable, and NA indicates an untested condition). Total HIV‐1 DNA is expressed as total HIV‐1 DNA copies/10^6^ CD8^+^‐depleted PBMCs. The cultures highlighted in gray showed a higher viral production with the LRA combination than with the corresponding LRAs alone, while the cultures highlighted in black were reactivated only by the combinatory treatment and not by the LRAs individually. Patients indicated by the # symbol are only reactivated by one or more combinations.

**Figure 6:**
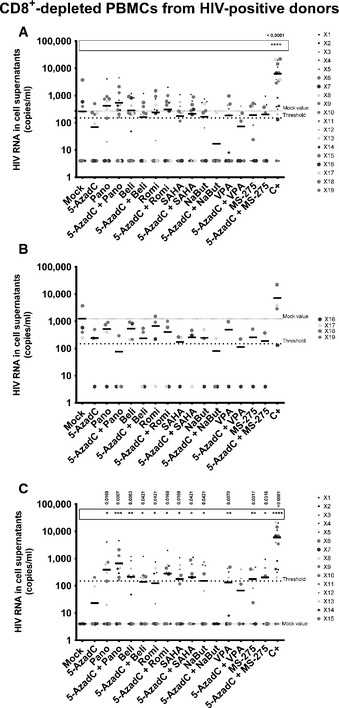
Representation of reactivation status of *ex vivo* cultures of CD8^+^‐depleted PBMCs isolated from HIV ^+^ patients A–CFrom data of *ex vivo* cultures of CD8^+^‐depleted PBMCs isolated from HIV ^+^ patients (Table 2), the extracellular HIV‐1 genomic RNA levels for each LRA treatment are represented from all patient cell cultures (A), from patient cell cultures presenting no viral reactivation in mock condition (C), and from patient cell cultures exhibiting reactivation in mock condition (B). One night after cell purification, cells were mock‐treated or treated with 5‐AzadC. Three days post‐treatment, 1/3 of medium was replaced and HDACIs were added to the cultures. Six days after 5‐AzadC treatment, the concentration of viral RNA in culture supernatants was determined (in copies/ml). The results were reported as the actual HIV RNA copy numbers/ml or as an estimated value calculated as 50% of the smallest value when HIV RNA was not detected in order to assign a log value. Means are represented. Nonparametric one‐way ANOVA for independent samples (Kruskal–Wallis) followed by paired comparisons between each treated condition and the mock‐treated condition (Mann–Whitney test) are performed.

We detected genomic viral RNA in the mock‐treated culture supernatant from 4 out of 19 patient cell cultures (X16‐X19, Table 2). This observation could be explained by the activation of HIV‐infected cells during the purification procedure. Global analysis of all 19 patient cell cultures showed that treatment with 5‐AzadC alone led to the reactivation of 21.1% of the patient cell cultures, a percentage similar to that obtained in the mock‐treated condition. In contrast, HDACIs alone increased the percentage of reactivated patient cell cultures (33.3–52.6% depending on the HDACI tested) (Table 2). Sequential combinatory treatments, except for the 5‐AzadC + NaBut and 5‐AzadC + VPA combinations, were more potent than the mock‐treated condition and led to the reactivation of 36.8–52.6% of cell cultures. Interestingly, only 5‐AzadC + panobinostat and 5‐AzadC + romidepsin combinations produced increases in percentage of reactivated patient cell cultures as compared to individual drug treatments (Table 2). These percentages, 52.6 and 42.1%, respectively, indicated ~2‐fold increases compared to the percentage obtained with the mock‐treated condition. Importantly, we also observed that, in some *ex vivo* patient cell cultures, some combined treatments caused higher levels of viral production than the levels observed after the individual treatments (Table 2, values highlighted in gray). Moreover, HIV‐1 recovery was only observed with some LRA combinations, but not with the corresponding individual LRAs (Table 2, values highlighted in black). Among these patient cell cultures presenting values in black, three cultures (X7, X11, and X14 indicated by a # symbol in Table 2) presented the reactivation of viral production exclusively after the sequential combined treatments: 5‐AzadC + romidepsin and 5‐AzadC + SAHA combinations for X7, 5‐AzadC + panobinostat combination for X11, and 5‐AzadC + panobinostat for X14. In terms of extracellular HIV RNA (Fig 6A), we observed that only the 5‐AzadC + panobinostat and 5‐AzadC + romidepsin combinations presented mean levels of extracellular viral RNA higher than the mean levels observed after the individual LRA treatments and higher than the mean level obtained in mock‐treated condition. However, the results including all patient cell cultures were not statistically relevant, except for the positive control condition (Fig 6A). Importantly, we observed that the 15 patient cell cultures presenting no viral activation in mock condition (patients X1–X15) clearly exhibited a more potent viral production following 5‐AzadC + panobinostat and 5‐AzadC + romidepsin treatments (see Table 2, gray and black boxes) in comparison with the last 4 patient cell cultures presenting viral reactivation in mock condition (patients X16–X19). Therefore, we decided to analyze separately the reactivation data from the two groups: (i) the four patient cell cultures exhibiting viral activation in mock‐treated condition (Fig 6B); and (ii) the 15 patient cell cultures presenting no viral reactivation in mock condition (Fig 6C).

When performing analysis for the first group including the four patient cell cultures presenting HIV‐1 recovery in mock‐treated condition (Fig 6B), we observed that all means of HIV extracellular RNA levels after the different treatments, except after the positive control, were lower than the mean observed in mock‐treated condition (Fig 6B).

For the second group, the reactivation profiles we obtained were similar to the reactivation profiles observed after global analysis of the 19 patient cell cultures (compare Fig 6C to Fig 6A). Interestingly, not only combinations 5‐AzadC + panobinostat and 5‐AzadC + romidepsin but also 5‐AzadC + SAHA and 5‐AzadC + MS‐275 produced higher mean levels of viral RNA than the mean levels obtained with the individual treatments (Fig 6C). Moreover, in contrast to what we observed for the 19 patient cell culture global analysis, we observed statistically relevant viral recoveries for the second group of 15 patient cell cultures (Fig 6C). Indeed, in this group, all individual treatments, except 5‐AzadC, produced statistically relevant increases in the mean levels of extracellular HIV‐1 RNA (Fig 6C). Moreover, all 5‐AzadC + HDACI combinations, except 5‐AzadC + NaBut and 5‐AzadC + VPA, produced statistically relevant increases in HIV recovery as compared to mock‐treated condition. Notably, the 5‐AzadC + panobinostat combined treatment was the most potent and the only one to produce highly statistically relevant increase (*P* = 0.0007) as compared to mock condition.

**Figure 7:**
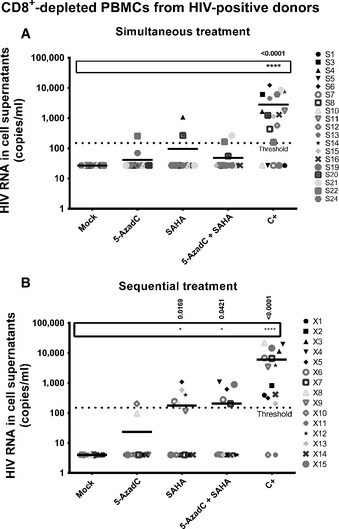
Comparison of simultaneous 5‐AzadC + SAHA combined treatment with its corresponding sequential treatment in *ex vivo* cultures of CD8^+^‐depleted PBMCs isolated from aviremic cART‐treated HIV ^+^ patients Reactivation status of patient cell cultures presenting no viral reactivation in mock condition isolated from aviremic cART‐treated HIV ^+^ patients (shown in Appendix Table S2).Reactivation status of patient cell cultures presenting no viral reactivation in mock condition isolated from aviremic cART‐treated HIV ^+^ patients (shown in Table 2).Data information: The results were reported as the actual HIV RNA copy numbers/ml or as an estimated value calculated as 50% of the smallest value when HIV RNA was not detected in order to assign a log value. Means are represented by a line. Nonparametric one‐way ANOVA for independent samples (Kruskal–Wallis) followed by paired comparisons between each treated condition and the mock‐treated condition (Mann–Whitney test) are performed.

Importantly, when we analyzed separately the *ex vivo* reactivation data shown in Fig 3E as we performed for Fig 6, we could clearly observe that the 5‐AzadC + SAHA combined treatment produced a beneficial effect on HIV‐1 reactivation when used sequentially as opposed to no viral reactivation for the corresponding simultaneous 5‐AzadC + SAHA treatment (compare Fig 7B with Fig 7A).

In conclusion, in agreement with our *in vitro* reactivation assays, we demonstrated *ex vivo* the importance of the sequential LRA treatments compared to their corresponding simultaneous treatments. Moreover, our data suggested that the 5‐AzadC + panobinostat and 5‐AzadC + romidepsin combinatory treatments could be more promising than the other combinations tested in order to reactivate HIV‐1 latency *in vivo*.

Among the cell types present in CD8^+^‐depleted PBMCs, latently infected resting CD4^+^ T cells represent the major reservoirs of HIV infection (Blankson *et al*, 2002; Kulkosky *et al*, 2004; Marcello, 2006). Three scenarios can produce latent HIV infection in resting CD4^+^ T cells: (i) productive infection of activated CD4^+^ T cells that survive long enough for the cell to transition to a resting memory state, (ii) infection of CD4^+^ T cells that are transitioning from an activated to a resting memory state, and (iii) direct infection of resting CD4^+^ T cell (Chavez *et al*, 2015). In order to assess in resting CD4^+^ T cells the effect of the two most promising combinations (5‐AzadC + panobinostat and 5‐AzadC + romidepsin), we purified HLA DR^−^ CD25^−^ CD69^−^ CD4^+^ T cells from the blood of 15 HIV‐1^+^ volunteer patients and we performed the sequential treatments described above.

**Figure 8:**
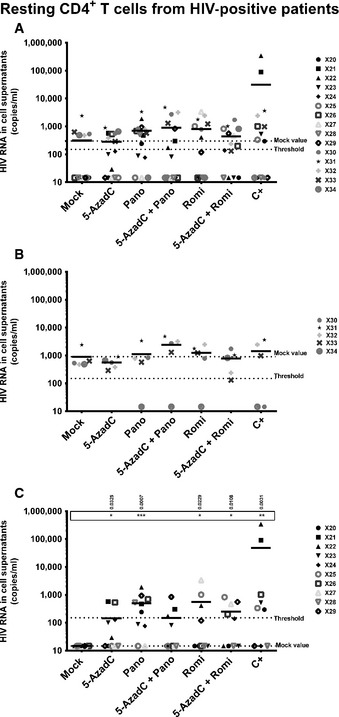
Representation of reactivation status of *ex vivo* cultures of resting CD4^+^ T cells A–CFrom data of *ex vivo* cultures of resting CD4^+^ T cells isolated from HIV ^+^ patients (Table 3), the extracellular HIV‐1 genomic RNA levels for each LRA treatment are represented from all patient cell cultures (A), from patient cell cultures presenting no viral reactivation in mock condition (C), and from patient cell cultures exhibiting reactivation in mock condition (B). One night after cell purification, cells were mock‐treated or treated with 5‐AzadC. Three days post‐treatment, 1/3 of medium was replaced and HDACIs were added in the cultures. Six days after 5‐AzadC treatment, the concentration of viral RNA in culture supernatants was determined (in copies/ml). The results were reported as the actual HIV RNA copy numbers/ml or as an estimated value calculated as 50% of the smallest value when HIV RNA was not detected in order to assign a log value. Means are represented. Nonparametric one‐way ANOVA for independent samples (Kruskal–Wallis) followed by paired comparisons between each treated condition and the mock‐treated condition (Mann–Whitney test) are performed.

In 5 out of the 15 patient cell cultures, genomic viral RNA was detected in mock‐treated condition (X30–X34, Table 3). Treatment with 5‐AzadC alone increased the percentage of reactivated patient cell cultures in a more important manner than the mock‐treated condition in resting CD4^+^ T‐cell cultures (Table 3), whereas this was not observed in CD8^+^‐depleted PBMC cultures (Table 2). Treatments with panobinostat or romidepsin alone were more potent than the mock‐treated condition and led to percentages of 66.7 and 50.0% of reactivated patient cell cultures, respectively. These percentages were close to the percentage observed with the positive control (64.3%) (Table 3). In contrast, their mean levels of viral RNA were lower than the mean level observed with the positive control, suggesting that these HDACIs alone activated HIV in numerous patient cell cultures but to a weaker extent than the positive control (Fig 8A). The percentage of reactivated patient cell cultures observed with the 5‐AzadC + panobinostat combination was lower than the percentages obtained with 5‐AzadC or panobinostat alone (Table 3). However, the mean level of HIV RNA induced by the 5‐AzadC + panobinostat combination was slightly higher than the mean levels observed with the corresponding LRAs alone (Fig 8A). In contrast, the 5‐AzadC + romidepsin treatment led to a small increase in percentage of reactivated patient cell cultures (Table 3) and induced a weaker HIV RNA mean level than the drugs used alone (Fig 8A). However, the results including all patient cell cultures were not statistically relevant. Importantly, we also observed that, in some *ex vivo* patient cell cultures, both combinatory treatments allowed higher levels of viral production than the levels observed with the individual treatments (Table 3, values highlighted in gray). As performed for the CD8^+^‐depleted PBMCs, we decided to analyze separately the reactivation data for (i) the group of 5 patient cell cultures exhibiting viral activation in mock condition (Fig 8B) and (ii) the group of 10 patient cell cultures presenting no viral reactivation in mock condition (Fig 8C). Interestingly, as shown in Fig 8B, the first group of patient cell cultures exhibited higher viral production following 5‐AzadC + panobinostat treatment as compared to the individual panobinostat treatment, suggesting that cells from these patients might have been weakly reactivated during purification, but remained responsive to some of the LRAs we used. In the second group of patient cell cultures, we observed that panobinostat and romidepsin alone presented higher and statistically relevant means of extracellular HIV RNA level than the means observed with the 5‐AzadC + panobinostat and 5‐AzadC + romidepsin combinations (Fig 8C). The differences in the effect of the combinations observed between the patient cell cultures which presented viral reactivation in mock condition (1^st^ group) and the patient cell cultures presenting no viral recovery in mock condition (2^nd^ group) could result from differences in cellular division status of reactivated cells and consequently differences in 5‐AzadC DNA incorporation.

**Table 3:**
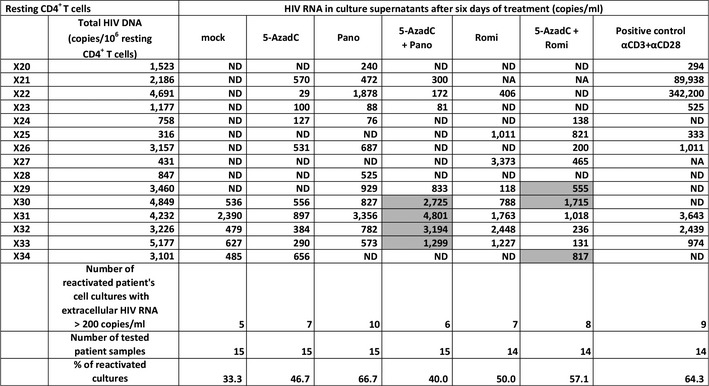
Representation of reactivation status of *ex vivo* cultures of HLA DR^−^ CD69^−^ CD25^−^ CD4^+^ T cells isolated from HIV^+^ patients

One night after cell purification, cells were mock‐treated or treated with 5‐AzadC. Three days post‐treatment, 1/3 of medium was removed and HDACIs were added in the cultures. Six days after 5‐AzadC treatment, the concentration of viral RNA in culture supernatants was determined (in copies/ml; ND means undetectable, and NA indicates an untested condition). Total HIV‐1 DNA is expressed as total HIV‐1 DNA copies/10^6^ resting CD4^+^ T cells. The cultures highlighted in gray showed a higher viral production with the LRA combination than with the corresponding LRAs alone.

Altogether, our results demonstrated *ex vivo* that a sequential treatment with 5‐AzadC + HDACI increased the reactivation potential of the HDACIs (principally panobinostat or romidepsin). Our data were less convincing in resting CD4^+^ T cells than in CD8^+^‐depleted PBMCs. However, it is important to note that the culture environment of the resting T cells was devoid of cytokines and growth factors produced by the other cell types present in the CD8^+^‐depleted PBMCS *ex vivo* environment, which might therefore be more representative of the *in vivo* situation in patients.

### Treatment with 5‐AzadC + panobinostat and 5‐AzadC + romidepsin does not induce global T‐cell activation and downregulates CD4 receptor expression on CD4^+^ T cells from HIV‐negative donors

**Figure 9:**
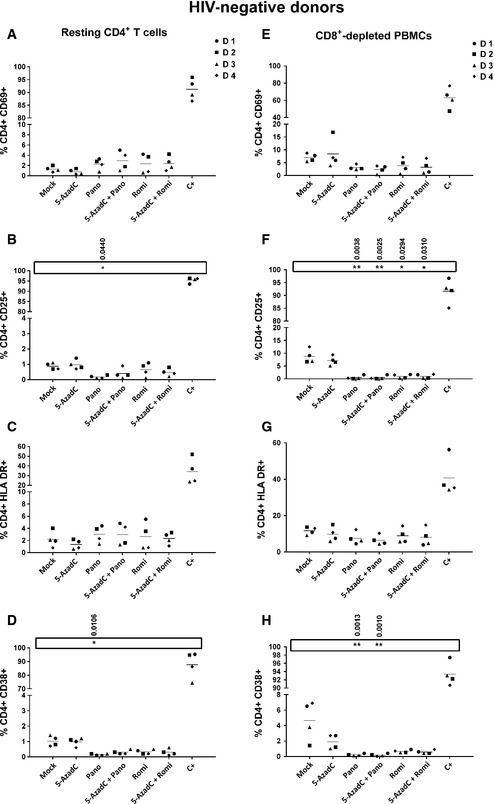
5‐AzadC + panobinostat and 5‐AzadC + romidepsin treatments do not induce global T‐cell activation A–HOne night after cell purification, CD8^+^‐depleted PBMCs (E–H) or HLA DR ^−^ CD69^−^ CD25^−^ CD4^+^ T cells (A–D) from 4 HIV‐negative donors were mock‐treated or treated with 5‐AzadC. Three days post‐treatment, 1/3 of medium was replaced and HDACIs were added to the cultures. The activation status of CD4^+^ T‐cell subset was assessed 6 days after 5‐AzadC treatment by flow cytometry analysis of cellular activation markers relative to mock treatment before 5‐AzadC stimulation corresponding to day 0. Means are represented. Nonparametric one‐way ANOVA for independent samples (Kruskal–Wallis) followed by paired comparisons between each treated condition and the mock‐treated condition (Mann–Whitney test) are performed.

LRAs that would be suitable for therapeutic use *in vivo* should not lead to non‐specific, long‐term, and robust immune activation. Additionally, drug‐mediated decrease in surface expression of CD4 receptor may be an important factor in the blockade of *de novo* HIV‐1 infection. To determine whether the two promising combinations described above activate immune cells, CD8^+^‐depleted PBMCs and HLA DR^−^ CD25^−^ CD69^−^ CD4^+^ T cells were purified from the blood of four HIV‐negative donors and treated with LRAs according to the sequential time schedule. The activation status of CD4^+^ T cells was assessed 6 days after LRA treatments by flow cytometry analysis of the cell surface activation markers and compared to mock treatment corresponding to day 0, before 5‐AzadC stimulation. As shown in Fig 9, no treatment, except for the positive control, increased in a statistically relevant manner the mean expression of the cell surface activation markers such as CD69 (early activation marker), CD25 (intermediate activation marker), HLA DR (late activation marker), and CD38 (late activation marker and predictive marker of HIV‐1 progression) in the two cell populations. Interestingly, in CD8^+^‐depleted PBMCs (cellular population including activated CD4^+^ T cells), we observed that panobinostat and romidepsin alone or in combination with 5‐AzadC induced a statistically relevant decrease in CD25^+^ CD4^+^ cells. Panobinostat alone or in combination with 5‐AzadC also induced a statistically relevant decrease in CD38^+^ CD4^+^ cells. These results were observed only with the two promising combinations (Fig 9) and not with the other combinations (Appendix Fig S4).

**Figure 10:**
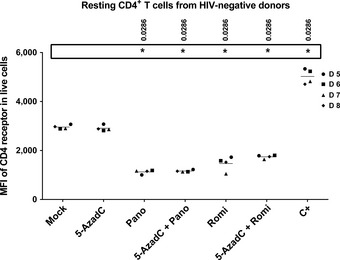
5‐AzadC + panobinostat and 5‐AzadC + romidepsin treatments induce a significant decrease in the cell surface CD4 receptor expression One night after cell purification, HLA DR ^−^ CD69^−^ CD25^−^ CD4^+^ T cells from 4 HIV‐negative donors were mock‐treated or treated with 5‐AzadC. Three days post‐treatment, 1/3 of medium was replaced and HDACIs were added to the cultures. The median fluorescence intensity of CD4 receptor of viable CD4^+^ T‐cell subset was assessed 6 days after 5‐AzadC treatment by flow cytometry analysis relative to mock treatment before 5‐AzadC stimulation corresponding to day 0. Means are represented. Nonparametric one‐way ANOVA for independent samples (Kruskal–Wallis) followed by paired comparisons between each treated condition and the mock‐treated condition (Mann–Whitney test) are performed.

In parallel, we evaluated whether the different treatments led to downregulation of the CD4 receptor. As shown in Fig 10, panobinostat and romidepsin alone or in combination with 5‐AzadC induced statistically relevant decreases in CD4 receptor expression on the cell surface. This would limit the number of HIV‐1 target cells. These results were observed only with the two promising combinations (Fig 10) and not with the other combinations (Appendix Fig S5).

Altogether, we concluded that 5‐AzadC + panobinostat and 5‐AzadC + romidepsin treatments did not induce global T‐cell activation and were able to decrease the activated status of CD4^+^ T cells. Moreover, we observed a negative regulation of the cell surface CD4 receptor expression after treatment with these combinations, suggesting an ability to limit HIV‐1 dissemination.

## Discussion

In this report, we assessed *in vitro* and *ex vivo* the HIV‐1 reactivation potential of combinations including demethylating agents and HDACIs at clinically tolerable concentrations. We showed that the DNA methylation inhibitor 5‐AzadC alone, but not 5‐AzaC, induced HIV‐1 expression after 72 h of treatment in a latently infected T‐cell line. Consequently, Dacogen (5‐AzadC), but not Vidaza (5‐AzaC), used at concentrations below human usual dosage, could be promising in combinatory treatments with other LRAs in strategies aimed at reducing the HIV‐1 reservoir size. After determination for each selected HDACI (VPA, NaBut, MS‐275, SAHA, belinostat, panobinostat, and romidepsin) of an optimal concentration in terms of its HIV‐1 reactivation potential in a latently infected T‐cell line and of its human tolerable *C*_max_, we demonstrated that a sequential 5‐AzadC + HDACI (except MS‐275) treatment synergistically induced HIV‐1 expression at both viral RNA and protein levels in two latently infected T‐cell lines. Our data highlighted for the first time that, in addition to the setup of LRA concentrations, the sequential time schedule of LRA combined treatments was very important to reach these synergistic activations of HIV production. Our results showed that the 5‐AzadC + panobinostat and 5‐AzadC + romidepsin combinations were more potent than the other combinations we tested, highlighting potential therapeutic implications for strategies aimed at reducing the pool of HIV‐1 latent reservoirs in cART‐treated patients. Next, we addressed the physiological relevance of our results using *ex vivo* cultures of CD8^+^‐depleted PBMCs and of resting CD4^+^ T cells isolated from cART‐treated aviremic HIV‐1^+^ patients. In both types of cell cultures, we observed that HDACIs alone were more potent in inducing HIV‐1 recovery than 5‐AzadC alone. Recent studies have failed to observe increased HIV particle production following *ex vivo* treatment with SAHA or romidepsin (Bullen *et al*, 2014; Cillo *et al*, 2014; Mohammadi *et al*, 2014). This discrepancy with the present study might result from the fact that we analyzed viral production using a very sensitive technique, the quantification of genomic HIV RNA in cell culture supernatants, as performed by Wei and colleagues who, in agreement with our data, have also observed a romidepsin‐ and SAHA‐induced HIV RNA release in resting CD4^+^ T‐cell cultures (Wei *et al*, 2014). Importantly, in agreement with our *in vitro* reactivation assays, we demonstrated *ex vivo* the importance of the treatment sequential time schedule for LRA combinations compared to the corresponding simultaneous combinatory treatments. This phenomenon could be explained by the fact that, in eukaryotic cell gene regulation, DNA methylation may function as a first control level that locks gene transcriptional activity (reviewed in Jones, 1985). In some systems, such as myogenesis or thymidine kinase induction, no stimulus other than demethylation is necessary for gene expression. However, in other systems, gene activation occurs in response to a cascade of events. For several genes whose activation requires 5‐AzaC as a primary stimulus and another inducer as a secondary stimulus, no significant response is observed with either stimulus alone (Jones, 1985). In the present study, our data suggest that the 5‐AzadC + panobinostat and 5‐AzadC + romidepsin sequential combinatory treatments could be more promising than the other combinations we tested in order to reactivate HIV‐1 latency *in vivo*.

Nevertheless, we observed that *ex vivo* viral production levels were weaker than the levels observed in our previous studies using HMTIs, HDACIs, P‐TEFb‐releasing agents, or NF‐κB inducers (Reuse *et al*, 2009; Bouchat *et al*, 2012; Darcis *et al*, 2015). Of note, on the one hand, in these previous studies, we used doses inducing maximal HIV‐1 viral production, whereas in contrast, in the present report, we used LRA concentrations corresponding to compromises between potent HIV‐1 production and human tolerable *C*_max_. For example, panobinostat and romidepsin were used at concentrations 4‐ to 9.3‐fold and 6.4‐fold lower than human *C*_max_, respectively. On the other hand, 5‐AzadC was reported as a compound exhibiting HIV‐1 antiretroviral effects. Indeed, Bouchard *et al* have reported that 5‐AzadC at the dose we used is able to completely inhibit HIV‐1 replication when the drug is added to the cell medium at least 2 h before infection (Bouchard *et al*, 1990). More recently, a study has validated these latter results in a mouse model (murine acquired immunodeficiency syndrome; MAIDS), where 5‐AzadC alone or in combination with gemcitabine decreases HIV‐1 replication through the introduction of lethal mutations during viral reverse transcription, leading to a decrease in latent reservoir pool and in disease progression (Clouser *et al*, 2012). Targeting reverse transcription, 5‐AzadC has been compared to AZT and presents a higher inhibition effect than AZT at equimolar amounts (Bouchard *et al*, 1990). Importantly, the antiviral activity of 5‐AzadC is also comparable to that of tenofovir and raltegravir in the case of feline leukemia virus (Greggs *et al*, 2012). Of note, in our *ex vivo* patient cell cultures, we chose to work in the absence of antiretroviral compounds (i) to amplify viral production, thereby facilitating measurement of cell culture reactivation following LRA treatments, and (ii) to allow plating less cells per well, thereby allowing testing a larger number of different LRA treatments with the blood sample of a single patient. Therefore, viral productions we observed here could likely result from either reactivation of HIV‐1 gene expression in latent cells or viral production in cells newly infected by the neosynthesized viruses. Nevertheless, by treating cells with 5‐AzadC, which has been reported to exhibit an antiretroviral activity (Bouchard *et al*, 1990), we probably only measured HIV production from reactivated latent cells. Indeed, the Bouchard *et al*'s study has shown that, in patient cells, 5‐AzadC, at the same dose than the one we used here, limits the infection of the cells to one replication cycle (Bouchard *et al*, 1990). This phenomenon could explain, at least in part, the fact that in this report some patient cell cultures presented a level of viral production following treatment with HDACIs alone higher than the level observed following the corresponding 5‐AzadC + HDACI combinatory treatment.

The beneficial effect of the two combinations selected here (5‐AzadC + panobinostat and 5‐AzadC + romidepsin) could be explained by the cooperation between the two targeted epigenetic mechanisms, but also by intrinsic properties of the HDACIs we used. Firstly, panobinostat is likely the most potent pan‐HDAC inhibitor in clinical development (Prince *et al*, 2009). The synergistic effect of 5‐AzadC in combination with panobinostat could result from the inhibition of DNA methyltransferase (DNMT) activity, since a downregulation of DNMT mRNAs and protein levels after panobinostat treatment has been reported (Zopf *et al*, 2012). Moreover, another study has shown that panobinostat treatment depletes DNMT1 and the HMT EZH2 protein levels and disrupts the interaction of DNMT1 with EZH2 (Fiskus *et al*, 2009), an HMT implicated in HIV‐1 transcriptional repression (Friedman *et al*, 2011). Secondly, romidepsin presents a unique intracellular pharmacology (Furumai *et al*, 2002). Moreover, synergistic activation observed with 5‐AzadC + romidepsin could be due to the fact that romidepsin belongs to the depsipeptide class of HDACIs. Indeed, a previous study has shown that depsipeptide exhibits a significant demethylating activity on the promoters of several genes (Wu *et al*, 2008). Depsipeptide also suppresses the expression of the HMTs G9A and SUV39H1, which in turn results in a decrease in di‐ and trimethylated H3K9 around these gene promoters. Therefore, romidepsin not only interferes with histone acetylation but also with two other epigenetic marks (di‐ and trimethylation of H3K9) previously demonstrated as involved in HIV‐1 latency (du Chene *et al*, 2007; Marban *et al*, 2007; Imai *et al*, 2010).

In a therapeutic goal, the ideal compounds should not lead to non‐specific, long‐term, and robust immune activation. In this context, we observed that 5‐AzadC + panobinostat and 5‐AzadC + romidepsin treatment did not induce global T‐cell activation and were able to decrease the activation level of activated CD4^+^ T cells. Interestingly, we reported a negative regulation of the cell surface CD4 receptor expression after treatment by these combinations, suggesting a limitation of HIV‐1 target cells after LRA treatment and an obstacle for virus dissemination.

Despite promising aspects of these combinations, one concern might be that 5‐AzadC needs to be incorporated into DNA. However, the pool of latently infected memory CD4^+^ T cells is maintained throughout patient life by homeostatic proliferation of memory T cells and/or intermittent antigen‐driven clonal expansion (Chomont *et al*, 2009). Moreover, two recent studies suggest that HIV integrates preferentially into cancer‐associated genes and other genes that promote cell proliferation in a manner distinct from homeostatic proliferation (Maldarelli *et al*, 2014; Wagner *et al*, 2014). Consequently, in these integration cases, HIV‐1‐infected T cells would divide faster than uninfected T cells. Finally, it should be noted that 5‐AzadC could exhibit activities independent of its DNA methylation inhibitory effect. Indeed, during the writing of this manuscript, Peterlin and colleagues have elegantly demonstrated that 5‐AzaC (at 10 μM) releases P‐TEFb from its inactive complex similar to known P‐TEFb‐releasing agents (Fujinaga *et al*, 2015). Consequently, 5‐AzadC could also have an HIV‐1 anti‐latency effect independently of its DNA incorporation.

In the race for a HIV cure, the levels of HIV‐1 reservoirs appear as one of the major factors influencing the duration of a remission after cART cessation. Reactivation of HIV gene expression in latently infected cells together with an efficient or intensive cART could serve as an inducible adjuvant therapy aimed at eliminating/decreasing the pool of latent viral reservoirs. Several clinical trials have started with HDACIs alone. However, these studies question the efficiency of these drugs used alone to induce a sufficient HIV‐1 production level and to reduce the viral reservoir size. Our results suggest that the use of 5‐AzadC in combination with HDACI could improve HIV‐1 reactivation in such anti‐latency clinical trials. However, it has been reported that some HDACIs (SAHA, panobinostat, and romidepsin) decrease the antiviral immune system functions (Jones *et al*, 2014). Notably though, these side effects were observed at higher doses than those reported in the literature in anti‐latency clinical trials using these HDACIs. Indeed, immunomodulatory effects of HDACI have been widely studied in the context of anticancer therapies. Undoubtedly, these effects will vary depending on the specific pathological state since the effects of an HDACI can be cell‐, tissue‐, or context‐dependent and can involve the modulation of specific inflammatory signaling pathways as well as epigenetic mechanisms (Akimova *et al*, 2012; Fairlie & Sweet, 2012). Overall, anti‐inflammatory effects of HDACI *in vivo* tend to target pathologic inflammatory responses but preserve normal immune cell functions (Akimova *et al*, 2012). For example, in the particular context of neoplasia, HDACIs prevent the ability of tumors to evade the immune system by enhancing host immunosurveillance and inducing appropriate local immune effector responses (Licciardi & Karagiannis, 2012). In the context of HIV infection, Olesen *et al* (2015) have reported using data from two recent separate clinical trials that the treatment of HIV‐1‐infected patients with panobinostat and romidepsin did not decrease the levels of HIV‐1‐specific effector memory CD8^+^ T cells. However, even if HDACIs seem to preserve immune function, it is likely that anti‐HIV latency purging strategies will have to be complemented with immunomodulatory approaches in order to clear reactivated infected cells. Therefore, our results are promising in the view of increasing viral production but if these combinations are tested in clinical trials, a stimulation of the immune response should probably be included in order to offset the effect of HDACIs. Nevertheless, studies in cell lines revealed that cells expressing HIV upon HDACI induction are more likely to undergo apoptosis than cells not expressing HIV (Shehu‐Xhilaga *et al*, 2009), suggesting that HDACIs could display features of selective killing of HIV expressing cells.

Altogether, our data showed that a sequential treatment with 5‐AzadC + HDACI may increase the reactivation potential of some HDACIs (principally panobinostat and romidepsin) and emphasized the importance of considering the use of demethylating agents in strategies to purge latent HIV reservoirs in some patients for whom other LRAs would be inefficient. Interestingly, three clinical trials for anticancer therapy in humans are ongoing with 5‐AzadC in combination with panobinostat (ClinicalTrial.gov NCT00691938, NCT01194908, and NCT00925132) and two trials with 5‐AzadC + romidepsin (ClinicalTrial.gov NCT00037817 and NCT00114257). Some of these clinical trials apply a sequential treatment. Consequently, the determination of the maximal tolerated dose and dose limiting toxicity, the safety, and the tolerability of administration schedule of these combinations in patients have already been studied in the context of anticancer therapies. Nevertheless, we and others have observed an important diversity between patients in terms of pattern of response to different LRAs. This phenomenon reflects the heterogeneity of the reservoirs and the multiplicity of the mechanisms which underlie latency and likely vary from one patient to the other. These observations highlight the necessity to test a great number of HIV‐1 patients in order to understand this heterogeneity. Our data also underline the importance to demonstrate LRA efficacy *ex vivo* before potential administration of these LRAs *in vivo* in clinical trials.

## Materials and Methods

### Reagents

5‐AzadC, NaBut, VPA, and SAHA (vorinostat) were purchased from Sigma Aldrich. MS‐275 was purchased from Enzo Life Sciences. Belinostat, panobinostat, and romidepsin were purchased from Selleckchem.

### Cell lines and cell culture

The T‐lymphoid cell lines Jurkat and J‐Lat (Jordan *et al*, 2003) were obtained and used as described in Quivy *et al* (2002) and Reuse *et al* (2009). These cell lines were grown in RPMI 1640 medium (Gibco‐BRL) supplemented with 10% fetal bovine serum, 50 U/ml of penicillin, 50 μg of streptomycin at 37°C in a humidified 95% air/5% CO_2_ atmosphere.

### Virus production assays

HIV‐1 production was measured in supernatants of the J‐Lat 8.4 and J‐Lat 15.4 cell cultures by the INNOTEST^®^ HIV Antigen mAb kit according to the manufacturer's instructions (Innogenetics). INNOTEST^®^ HIV Antigen mAb is an enzyme immunoassay for the detection of HIV p24 antigen in human serum, plasma, and cell culture supernatant.

### Metabolic activity assays

Metabolic activity was evaluated by the colorimetric test WST‐1 according to the manufacturer's instructions (Roche). The Cell Proliferation Reagent WST‐1, based on its cleavage to a soluble formazan depending on the glycolytic production of NAD(P)H in viable cells, is used for the spectrophotometric quantification of cell proliferation and viability in cell populations. Therefore, the amount of formazan dye formed directly correlates with the number of metabolically active cells in the culture.

### Flow cytometry

In the J‐Lat cell lines, transcriptional activation of the latent provirus can be detected in individual cells by flow cytometry since these cells harbor full‐length latent HIV‐1 provirus containing *gfp* gene in place of *nef*. These J‐Lat cell lines were mock‐treated or treated as indicated. FACS experiments were directly performed after cell fixation as previously described in Reuse *et al* (2009).

### RNA extraction and analysis of HIV‐1 transcripts

Total RNA samples were isolated using the RNeasy Plus kit (Qiagen) and digested with TURBO DNase (TURBO DNA‐free^™^ kit, Ambion). Retrotranscription was performed with Superscript III (Invitrogen). Initiated and elongated transcripts were detected with primers amplifying the TAR region (5′‐GTTAGACCAGATCTGAGCCT‐3′ and 5′‐GTGGGTTCCCTAGTTAGCCA‐3′) and primers amplifying *tat* (5′‐ACTCGACAGAGGAGAGCAAG‐3′ and 5′‐GAGATCTGACTGTTCTGATGA‐3′), respectively. cDNAs were quantified and normalized to the β‐actin mRNA level (5′‐GTCGACAACGGCTCCGGC‐3′ and 5′‐GGTGTGGTGCCAGATTTTCT‐3′).

### Study subjects

We selected 58 HIV^+^ volunteer treated patients at the St‐Pierre Hospital (Belgium) with undetectable viral load (< 50 HIV‐1 RNA copies/ml of plasma since at least 1 year), under cART (for at least 1 year), and with a CD4^+^ T‐cell level superior to 300 cells/mm^3^ of blood. Characteristics of these patients were well documented and presented in Appendix Table S4. Ethical approval was granted by the Human Subject Ethics Committees of hospital. All patients enrolled in the study provided written informed consent for donating blood. The blood samples from HIV‐negative donors were obtained from the transfusion center of Charleroi (Belgium). The experiments concerning patient cells are conformed to the principles set out in the WMA Declaration of Helsinki and the Department of Health and Human Services Belmont report.

### Isolation of CD8^+^‐depleted PBMCs and resting CD4^+^ T cells

CD8^+^‐depleted PBMCs used in reactivation assays were isolated from the fresh whole blood of HIV^+^ patients as previously described (Reuse *et al*, 2009). Resting CD4^+^ T cells (HLA DR^−^ CD69^−^ CD25^−^ CD4^+^) were isolated using a negative selection by an automate RoboSep (StemCell Technologies). PBMCs isolated by density centrifugation on a Ficoll–Hypaque gradient (Pharmacia) were washed with NaCl 0.9%, counted, and placed on RoboSep according to the manufacturer's instructions. CD8^+^‐depleted PBMCs or HLA DR^−^ CD69^−^ CD25^−^ CD4^+^ T cells were seeded in LGM‐3 growth medium (Lonza) at 2 × 10^6^ cells/ml (6 × 10^6^ cells/well) and 0.8 × 10^6^ cells/ml (2.5 × 10^5^ cells/well), respectively. One night after isolation, cells were mock‐treated or treated with anti‐CD2 + anti‐CD28 (experiments performed in 2009) or anti‐CD3 + anti‐CD28 (experiments performed in 2014) antibodies as a positive control for global T‐cell activation (Pierres *et al*, 1988; Costello *et al*, 1993) or by 5‐AzadC or by the appropriate HDACI for simultaneous treatment. For sequential treatment, three days after 5‐AzadC treatment, 1/3 of medium was removed and replaced by medium containing the appropriate HDACI.

### Quantitative assessment of HIV‐1 RNA

Six days after treatment, culture supernatants were collected and HIV‐1 RNAs were extracted by QIAamp Viral RNA Mini Kit (Qiagen) according to the manufacturer's instructions. Samples were next tested for quantitative HIV‐1 RNA levels using the Generic HIV RNA cell kit based on RT–qPCR (Biocentric, Bandol, France) (detection limits of HIV‐1 RNA copies/ml depended on supernatant volumes collected for RNA quantification).

### Quantification of total HIV‐1 DNA

Total cellular DNA was extracted from patient cells after cell purification using either the DNA blood and tissue kit (Qiagen) or the QIAampDNA Micro kit (Qiagen) or QIAampDNA blood mini kit (Qiagen). Total HIV‐1 DNA was then quantified by the Generic HIV DNA cell kit based on qPCR [Biocentric, Bandol, France (Avettand‐Fenoel *et al*, 2009)].

### Analysis of T‐cell activation by flow cytometry

For T‐cell activation analysis, CD8^+^‐depleted PBMCs or resting CD4^+^ T cells isolated from the blood of uninfected donors were used to establish *ex vivo* cell cultures. Cells corresponding to day 0 and day 6 were collected and were stained with relevant antibodies. All antibodies were purchased from BD Biosciences and were included in two antibody cocktails. The first cocktail included anti‐CD4 (dilution 1:40, 557852), anti‐CD8 (dilution 1:10, 345774), and anti‐CD38 (dilution 1:10, 345806). The second cocktail included anti‐CD4 (dilution 1:10, 345770), anti‐CD8 (dilution 1:40, 348813), anti‐HLA DR (dilution 1:10, 347401), anti‐CD69 (dilution 1:10, 347823), and anti‐CD25 (dilution 1:40, 340907). Studies assessing CD4 receptor downregulation were performed in resting CD4^+^ T cells which were incubated with anti‐CD4 (dilution 1:10, 345770) and LIVE/DEAD Fixable Near‐IR Dead Cell Stain (Molecular Probes) in order to discriminate between viable and non‐viable cells. Median fluorescent intensity (MFI) of CD4 was then analyzed on viable cells. All experiments were assessed by flow cytometry analysis on a FACS Canto II (Becton‐Dickinson) and analyzed using the FACS Diva Software (Becton‐Dickinson). An arbitrary threshold was set using mock‐treated cells analyzed at day 0 for each HIV‐negative donor.

### Statistical analysis

The HIV‐positive donor selection was based on the three criteria described above. The HIV‐negative donor selection was based on unique criteria where no HIV infection is detected. The donor selection was randomized to avoid any personal or subjective bias. T‐cell activation data, *ex vivo* reactivation studies using patient cell cultures of CD8^+^‐depleted PBMCs or of resting CD4^+^ T cells are shown as means. Data sets were analyzed using a nonparametric one‐way ANOVA for independent samples (Kruskal–Wallis) followed by paired comparisons between each treated condition and the mock‐treated condition (Mann–Whitney test). The threshold of statistical significance α was set at 0.05. *P*‐value < 0.05 (*: *P* adjusted < 0.05, **: *P* adjusted < 0.01, ***: *P* adjusted < 0.001, ****: *P* adjusted < 0.0001) was considered statistically relevant. Analyses were performed using Prism version 6.0 (GraphPad software).

## Author contributions

CVL, CR, OR, and SB conceived and designed the experiments. SB, ND, AK, GD, BVD, J‐SG, CV, and MP performed the experiments. AM, VA‐F, ND, SB, and CR performed measurements of HIV‐1 genomic RNA concentrations. AM, VA‐F, and CR performed measurements of total HIV‐1 DNA concentrations. KK, SDW, and NC performed patient selection. AK and FC performed analyses of global T‐cell activation. SB and CVL wrote the paper.

## Conflict of interest

The authors declare that they have no conflict of interest.

**The paper explained**

**Problem**

More than 30 years after its discovery, HIV‐1 remains a major problem of public health. Persistence of truly latent (i.e. non‐defective) HIV‐1 proviruses represents a major obstacle to eradication. Indeed, the levels of HIV‐1 reservoirs appear as one of the critical factors influencing the duration of a remission after cART cessation. Consequently, a decline of the HIV‐1 latent reservoirs to a level sufficient to permit an efficient control of the infection by the host immune system might allow interruptions in therapy (“treatment‐free windows”). Reactivation of HIV gene expression in latently infected cells together with an efficient or intensified cART could serve as an adjuvant therapy aimed at eliminating/decreasing the pool of latent viral reservoirs. In this regard, results from HIV clinical trials using histone deacetylase inhibitors (HDACIs) question the efficiency of these drugs used alone and underline the need to evaluate other latency‐reversing agents (LRAs) in combination with HDACIs.

**Results**

In this report, we thoroughly studied the sequential administration of a DNA‐demethylating agent and clinically tolerable HDACIs first *in vitro* in latently infected T‐cell lines and next *ex vivo* in a large number (*n* = 58) of cART‐treated aviremic HIV‐1^+^ patient cell cultures. Interestingly, we showed that a sequential treatment with 5‐AzadC and HDACIs was more effective than the corresponding simultaneous treatment to observe a synergistic activation of HIV production *in vitro* and *ex vivo*. Moreover, only two of the sequential combinatory treatments tested induced HIV‐1 particle recovery in a higher manner than the LRAs alone in *ex vivo* patient cell cultures of CD8^+^‐depleted PBMCs. In conclusion, we reported for the first time that, in addition to concentration setup and combinatory aspect, the treatment sequential time schedule of LRA combination might be critical to design purging strategies aimed at decreasing the HIV‐1 reservoir size.

**Impact**

Our results based on a molecular virology view clearly illustrate a clinically focused translational research. We think that our study could interest and inspire HIV clinicians in order to elaborate clinical trial protocols. Indeed, this study clearly showed that the two promising combinations (5‐AzadC + panobinostat and 5‐AzadC + romidepsin) presented a reactivation potential at concentrations lower than the human tolerable plasmatic concentrations without presenting decreased cellular metabolic activity and global T‐cell activation. Consequently, our findings should be beneficial for the design of future anti‐latency therapeutic strategies and therefore constitute a step forward for HIV remission.

## Supporting information



Appendix

Review Process File
