# Supplementary material for: Sequential treatment with 5‐aza‐2′‐deoxycytidine and deacetylase inhibitors reactivates HIV‐1
Source: EMBO Mol Med. 2015 Dec 17;8(2):117–38. doi: 10.15252/emmm.201505557 (PMC4734845; doi:10.15252/emmm.201505557)
Supplement: Supplementary file 1 — Appendix [file EMMM-8-117-s001.pdf]

Table of content

Appendix Figure S1: The DNA methylation inhibitor 5-AzadC induces in a dose-dependent manner the MFI of GFP-positive latently-infected T cells.

Appendix Figure S2: Metabolic activity is similar in uninfected and in latently-infected cells after treatment by HDACIs.

Appendix Figure S3: Sequential 5-AzadC + HDACI treatments induce the MFI of GFP-positive latently-infected T cells.

Appendix Figure S4: 5-AzadC + HDACIs treatments do not induce global T-cell activation.

Appendix Figure S5: Only the 5-AzadC + Panobinostat and 5-AzadC + Romidepsin treatments induce significant decreases of the cell surface CD4 receptor expression.

Appendix Table S1: Representation of mean viral production, fold induction and fold synergy corresponding to Fig3A and 3B.

Appendix Table S2: Representation of reactivation status of *ex vivo* cultures of $CD8^+$-depleted PBMCs isolated from HIV^+ patients.

Appendix Table S3: Representation of mean viral production, fold induction and fold synergy corresponding to Fig4A and 4B.

Appendix Table S4: Representation of patients' characteristics

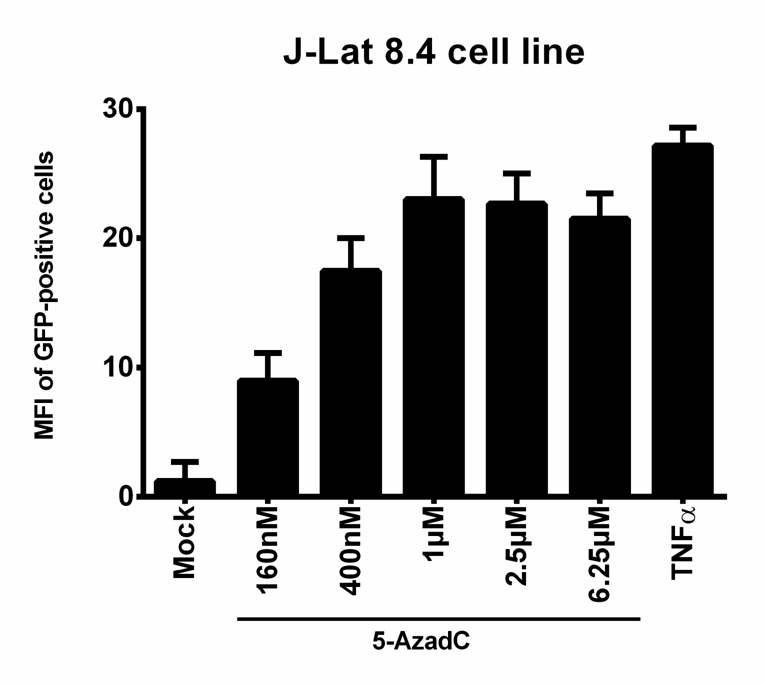

Appendix Figure S1: The DNA methylation inhibitor 5-AzadC induces in a dose-dependent manner the MFI of GFP-positive latently-infected T cells.
J-Lat 8.4 cell line was mock-treated or treated with increasing concentrations of 5-AzadC. At 72 h post-treatment, FACS analyses were performed and the median fluorescence intensity (MFI) of GFP^{-}positive cells was analysed. Means and standard errors of the means from three independent biological duplicates (n=6) are indicated.

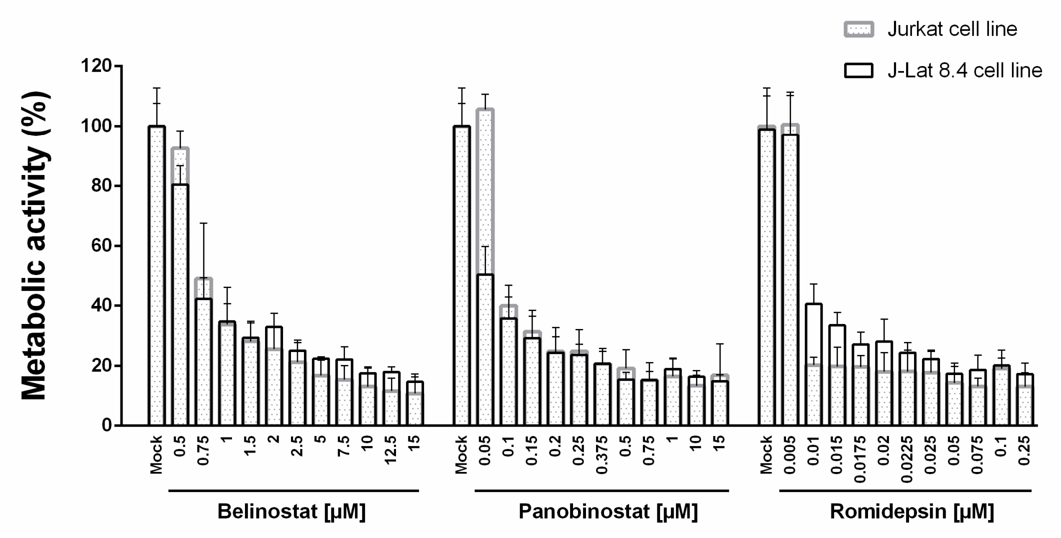

Appendix Figure S2: Metabolic activity is similar in uninfected and in latently-infected cells after treatment by HDACIs.
J-Lat 8.4 and Jurkat cells were mock-treated or treated with increasing concentrations of HDACIs. At 24 h post-treatment, metabolic activity was assessed by a WST-1 assay. Means and standard errors of the means from three independent biological duplicates (n=6) are indicated. The results obtained with mock-treated cells were arbitrarily set at a value of 100%.

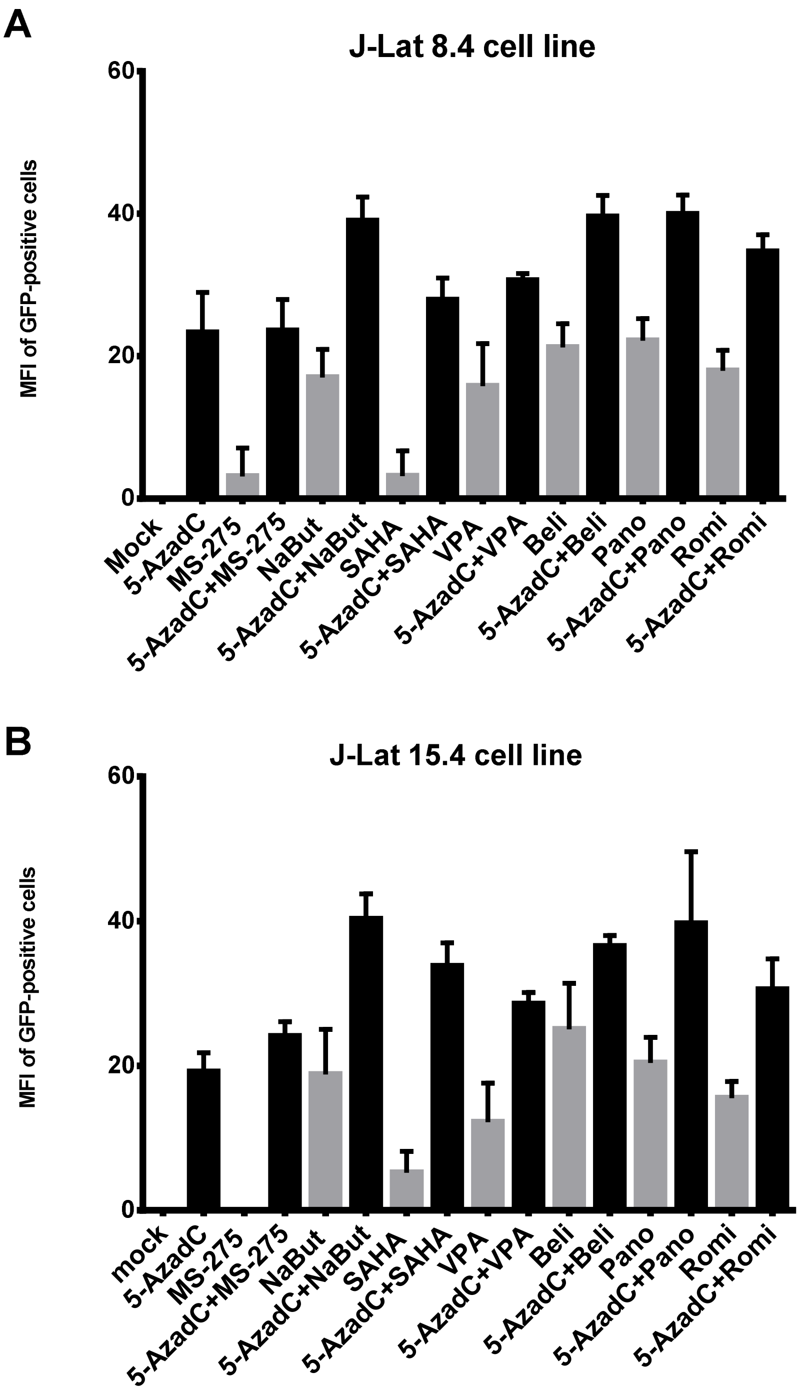

Appendix Figure S3: Sequential 5-AzadC + HDACI treatments induce the MFI of GFP-positive latently-infected T cells.

A-B J-Lat 8.4 (A) and J-Lat 15.4 (B) cells were mock-treated or treated with 5-AzadC. At 48 h post-treatment, HDACIs were then added for 24 h. At 72 h 5-AzadC post-treatment, FACS analyses were performed and the median fluorescence intensity (MFI) of GFP-positive cells was analyzed. Means and standard errors of the means from three independent biological duplicates (n=6) are indicated.

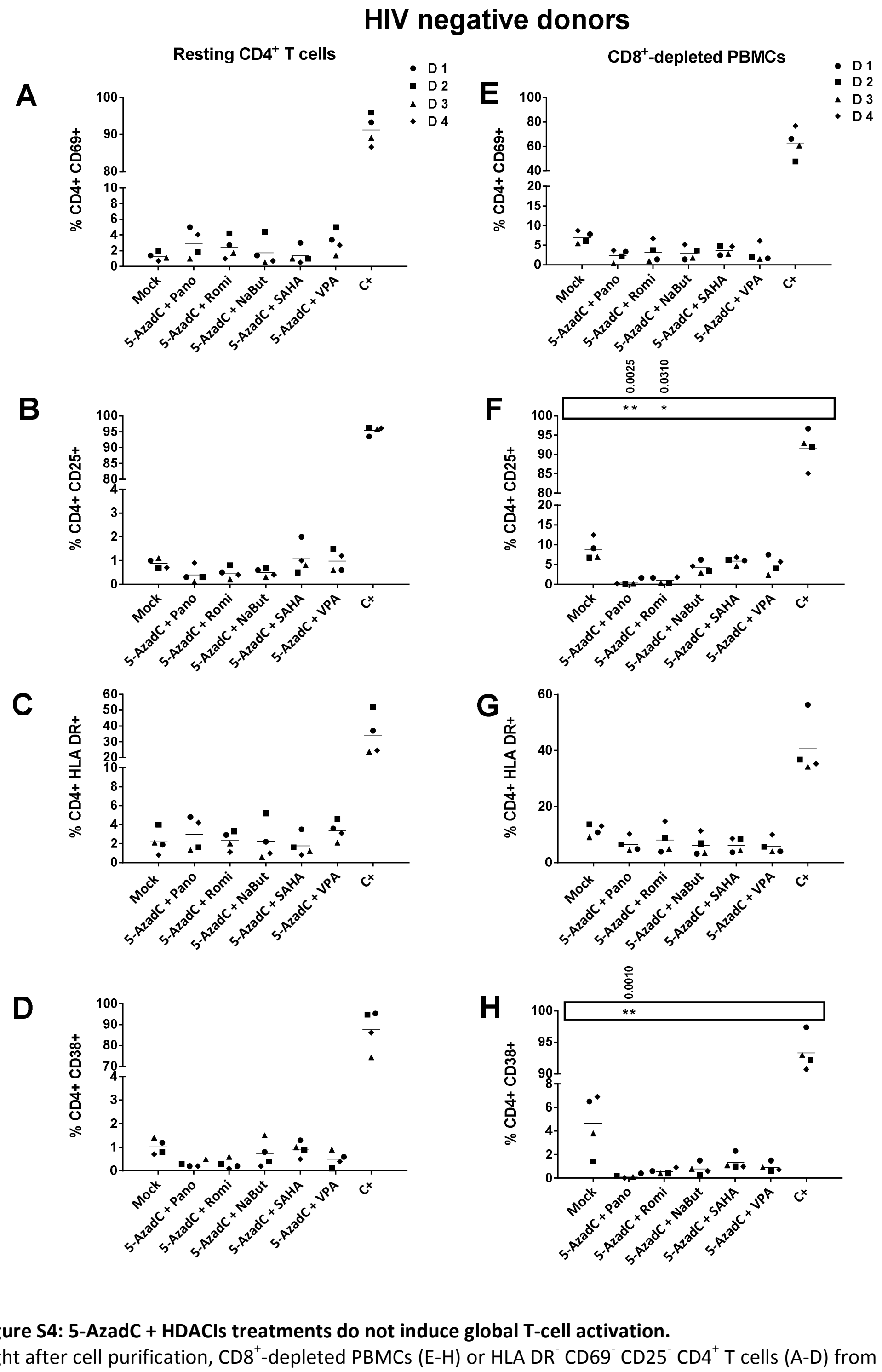

Appendix Figure S4: 5-AzadC + HDACIs treatments do not induce global T-cell activation.

A-H One night after cell purification, $CD8^+$-depleted PBMCs (E-H) or HLA DR^- $CD69^-$ $CD25^-$ $CD4^+$ T cells (A-D) from 4 HIV negative donors were mock-treated or treated with 5-AzadC. Three days post-treatment, 1/3 of medium was replaced and HDACIs were added in the cell cultures. The activation status of $CD4^+$ T cell subset was assessed 6 days after treatment by flow cytometry analysis of cellular activation markers relative to mock treatment before 5-AzadC stimulation corresponding to day 0. Data were represented as dot plots where means are represented by a line. Nonparametric one-way ANOVA for independent samples (Kruskal-Wallis) followed by paired comparisons between each treated condition and the mock-treated condition (Mann-Whitney test) are performed.

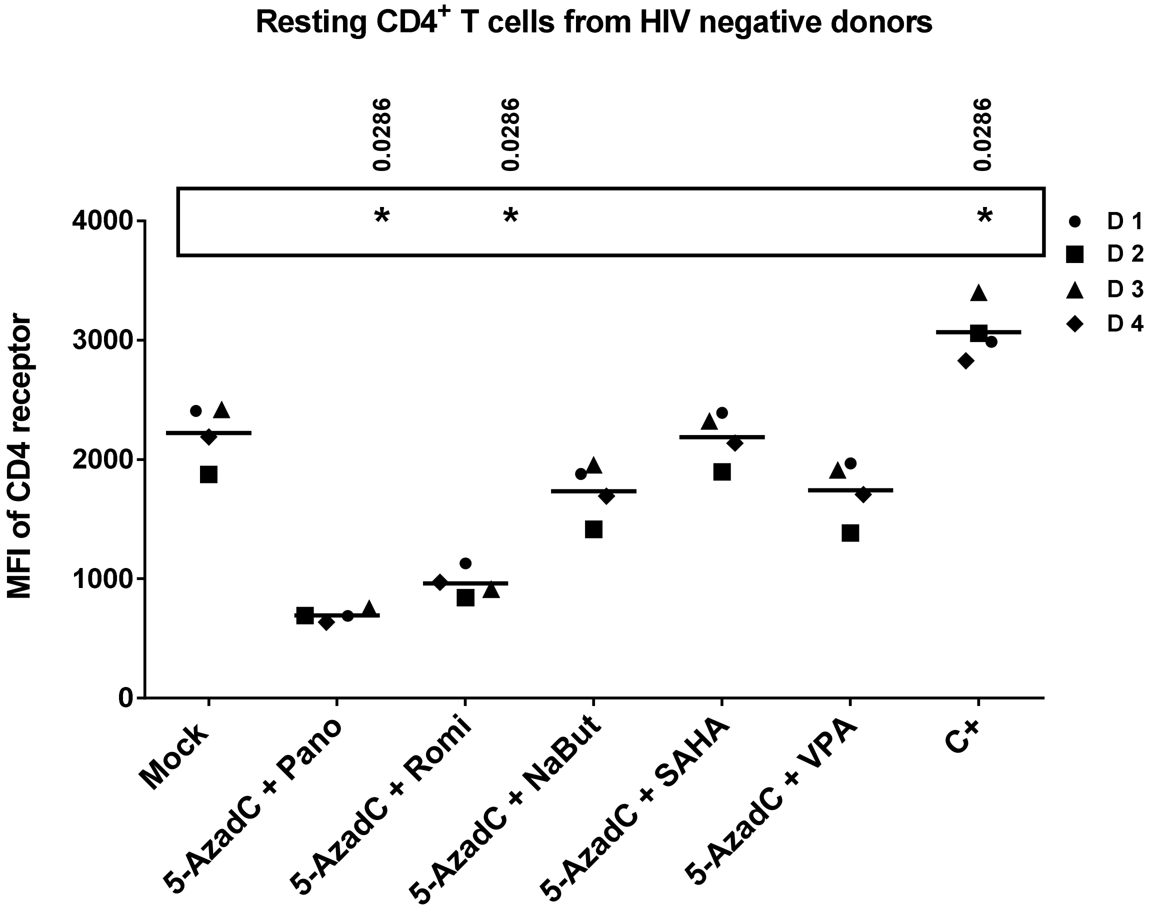

Appendix Figure S5: Only the 5-AzadC + Panobinostat and 5-AzadC + Romidepsin treatments induce significant decreases of the cell surface CD4 receptor expression.

One night after cell purification, HLA DR^- $CD69^-$ $CD25^-$ $CD4^+$ T cells from 4 HIV negative donors were mock-treated or treated with 5-AzadC. Three days post-treatment, 1/3 of medium was replaced and HDACIs were added in the cultures. The median fluorescence intensity (MFI) of CD4 receptor of total $CD4^+$ T cell subset was assessed 6 days after treatment by flow cytometry analysis relative to mock treatment before 5-AzadC stimulation corresponding to day 0. Data were represented by dot plot where means are presented with a line. Nonparametric one-way ANOVA for independent samples (Kruskal-Wallis) followed by paired comparisons between each treated condition and the mock-treated condition (Mann-Whitney test) are performed.

Appendix Table S1: Representation of mean viral production, fold induction and fold synergy corresponding to Fig3A and 3B.

	Mean of p24 levels (pg/ml)	Fold induction	Fold synergy
24 h of 5-AzadC including 24 h of SAHA treatment			
Mock	6.48	1.00	
5-AzadC	11.93	1.84	
SAHA	28.37	4.38	
5-AzadC + SAHA	21.52	3.32	0.53
48 h of 5-AzadC including 24 h of SAHA treatment			
Mock	8.49	1.00	
5-AzadC	9.94	1.17	
SAHA	6.86	0.81	
5-AzadC + SAHA	72.38	8.52	4.31
72 h of 5-AzadC including 24 h of SAHA treatment			
Mock	9.78	1.00	
5-AzadC	20.43	2.09	
SAHA	13.03	1.33	
5-AzadC + SAHA	101.83	10.41	3.04
72 h of 5-AzadC including 24 h of SAHA treatment			
Mock	10.20	1.00	
5-AzadC	19.45	1.91	
SAHA	13.57	1.33	
5-AzadC + SAHA	155.95	15.29	4.72
72 h of 5-AzadC including 48 h of SAHA treatment			
Mock	12.37	1.00	
5-AzadC	24.72	2.00	
SAHA	72.32	5.85	
5-AzadC + SAHA	444.48	35.93	4.58
72 h of 5-AzadC including 72 h of SAHA treatment			
Mock	10.41	1.00	
5-AzadC	26.85	2.58	
SAHA	64.40	6.19	
5-AzadC + SAHA	940.85	90.38	10.31

Appendix Table S2: Representation of reactivation status of *ex vivo* cultures of CD8^{+}-depleted PBMCs isolated from HIV^{+} patients.

CD8^{+}-depleted PBMCs	Concentration of HIV RNA in culture supernatants after six days of treatment (copies/ml)				
	**mock**	**5-AzadC**	**SAHA**	**5-AzadC + SAHA**	**Positive control αCD2+αCD28**
**S1**	**ND**	**ND**	**ND**	**ND**	**ND**
**S2**	**64**	**ND**	**330**	**316**	**1151**
**S3**	**ND**	**ND**	**ND**	**ND**	**6158**
**S4**	**ND**	**ND**	**1089**	**ND**	**1659**
**S5**	**ND**	**ND**	**ND**	**ND**	**ND**
**S6**	**ND**	**ND**	**ND**	**ND**	**12311**
**S7**	**ND**	**ND**	**ND**	**ND**	**ND**
**S8**	**ND**	**ND**	**ND**	**ND**	**436**
**S9**	**1116**	**ND**	**2291**	**ND**	**5027**
**S10**	**ND**	**ND**	**ND**	**ND**	**ND**
**S11**	**ND**	**ND**	**ND**	**ND**	**1733**
**S12**	**ND**	**ND**	**ND**	**ND**	**572**
**S13**	**ND**	**ND**	**ND**	**ND**	**4502**
**S14**	**ND**	**ND**	**ND**	**ND**	**7418**
**S15**	**ND**	**ND**	**ND**	**ND**	**1141**
**S16**	**ND**	**ND**	**ND**	**ND**	**1303**
**S17**	**573**	**54**	**ND**	**1508**	**1486**
**S18**	**75**	**ND**	**ND**	**90**	**568**
**S19**	**ND**	**ND**	**ND**	**ND**	**ND**
**S20**	**ND**	**ND**	**267**	**55**	**1237**
**S21**	**ND**	**ND**	**ND**	**261**	**8402**
**S22**	**ND**	**255**	**ND**	**164**	**159**
**S23**	**2803**	**ND**	**653**	**ND**	**ND**
**S24**	**ND**	**70**	**ND**	**ND**	**6074**
**Number of Reactivated patient's cell cutures with extracellular HIV RNA > 40 copies/ml**	**5**	**3**	**5**	**6**	**18**
**Number of tested patient samples**	**24**	**24**	**24**	**24**	**24**
**% of reactivated cultures**	**20.8**	**12.5**	**20.8**	**25.0**	**75.0**

One night after cell purification, CD8^{+}-depleted PBMCs were mock-treated or treated with 5-AzadC (1µM) and SAHA (1µM) alone or in combination as indicated. Six days after treatment, the concentration of viral RNA in culture supernatants was determined (in copies/ml; ND means undetectable). The cultures highlighted in gray showed a higher viral production with the LRA combination than with the corresponding LRAs alone, while the cultures highlighted in black were reactivated only by the combinatory treatment and not by the LRA individually.

Appendix Table S3: Representation of mean viral production, fold induction and fold synergy corresponding to Fig4A and 4B.

J-Lat 8.4	Mean of p24 levels (pg/ml)	Fold induction	Fold synergy
Mock	24.68	1.00	
5-AzadC	71.48	2.90	
MS-275	29.37	1.19	
5-AzadC + MS-275	117.24	4.75	1.16
NaBut	377.67	15.31	
5-AzadC + NaBut	5,140.81	208.34	11.45
SAHA	36.24	1.47	
5-AzadC + SAHA	1,552.86	62.93	14.42
VPA	208.67	8.46	
5-AzadC + VPA	2,139.30	86.70	7.64
Beli	390.41	15.82	
5-AzadC + Beli	9,751.18	395.18	21.11
Pano	2,221.01	90.01	
5-AzadC + Pano	16,083.85	651.82	7.02
Romi	2,510.18	101.73	
5-AzadC + Romi	10,996.88	445.67	4.26

J-Lat 15.4	Mean of p24 levels (pg/ml)	Fold induction	Fold synergy
Mock	12.85	1.00	
5-AzadC	147.94	11.51	
MS-275	12.70	0.99	
5-AzadC + MS-275	265.25	20.63	1.65
NaBut	46.43	3.61	
5-AzadC + NaBut	2,669.30	207.65	13.73
SAHA	10.31	0.80	
5-AzadC + SAHA	1,507.78	117.30	9.53
VPA	17.18	1.34	
5-AzadC + VPA	1,013.42	78.84	6.14
Beli	26.37	2.05	
5-AzadC + Beli	3,116.47	242.44	17.88
Pano	202.99	15.79	
5-AzadC + Pano	4,327.09	336.62	12.33
Romi	174.04	13.54	
5-AzadC + Romi	3,279.84	255.15	10.19

Appendix Table S4: Representation of patients' characteristics

Cell types	Patients	Year of birth	Year of HIV diagnostic	HIV subtype	$CD4^+$ Nadir	Year of 1st treatment	Year of undetectable status	Year of blood sampling	$CD4^+$ T count at last point	Last treatment	Year of last treatment administration
$CD8^+$-depleted PBMCs	S1	1951	2000	NA	NA	2001	2002	2009	857	KVX NVP	2006
	S2	1963	1988	NA	NA	1991	2008	2009	593	3TC DDI RTV FAPV MVC	2005
	S3	1945	2006	NA	NA	2006	2006	2009	550	CBV RTV FAPV	2006
	S4	1958	1995	NA	NA	1995	2004	2009	1328	DDI RTV ATV NVP	2004
	S5	1944	1986	NA	NA	1987	2008	2009	NA	TRU RTV ATV	2009
	S6	1957	2001	NA	NA	2001	2001	2009	585	AZT 3TC EFV	2005
	S7	1961	1998	NA	NA	1998	1998	2009	768	TZV	2002
	S8	1970	1992	NA	NA	1992	2003	2009	507	ABC TDF ATV	2003
	S9	1946	1988	NA	NA	1994	2007	2009	697	TRU RTV FAPV	2009
	S10	1960	1995	NA	NA	1997	2003	2009	706	TRU RTV FAPV	2009
	S11	1956	1995	NA	NA	1996	2002	2009	NA	TRU LPV	2009
	S12	1967	1995	NA	NA	1996	2001	2009	791	3TC TDF RTV ATV	2004
	S13	1963	1996	NA	NA	1996	2001	2009	1059	3TC TDF RTV FAPV	2005
	S14	1965	1996	NA	NA	1996	2007	2009	1089	TRU RTV FAPV	2009
	S15	1957	1991	NA	NA	1991	1999	2009	751	3TC ABC EFV	2004
	S16	1971	2001	NA	NA	2001	2002	2009	NA	TRU NVP	2009
	S17	1976	2006	NA	NA	2006	2006	2009	984	TRU LPV	2009
	S18	1986	1996	NA	NA	1996	2002	2009	930	TDF RTV ATV EFV	2004
	S19	1966	1996	NA	NA	1996	2003	2009	1303	AZT 3TC ABC	2000
	S20	1967	2006	NA	NA	2006	2007	2009	1047	TDF FTC ATC	2006
	S21	1977	2005	NA	NA	2005	2005	2009	607	TDF FTC EFV	2006
	S22	1955	2001	NA	NA	2005	2005	2009	770	3TC TDF EFV	2005
	S23	1947	1998	NA	NA	1998	2007	2009	881	RTV ATV	2000
	S24	1960	2000	NA	NA	2000	2006	2009	555	3TC TDF LPV NVP	2008
	X1	1950	1999	NA	290	2000	2000	2014	948	TZV	2002
	X2	1961	1997	B	224	1999	2009	2014	590	TRU ETV RLT	2011
	X3	1960	1997	NA	185	1997	2003	2014	428	ATR	2010
	X4	1963	2006	NA	256	2006	2006	2014	633	TRU ETV RLT	2009
	X5	1971	1997	D	255	1997	2009	2014	786	CBV RLT MVC	2009
	X6	1969	2011	F2	364	2012	2012	2014	723	KVX RTV ATV	2012
	X7	1969	1998	NA	138	1999	2012	2014	723	KVX RTV ATV	2012
	X8	1973	2005	NA	71	2005	2005	2014	513	TRU RTV FAPV	2009
	X9	1963	1996	NA	360	1996	1998	2014	777	KVX ATV	2014
	X10	1951	1990	NA	144	1991	1997	2014	629	TRU NVP	2014
	X11	1976	2005	NA	422	2012	2012	2014	603	TRU ATV RTV	2014
	X12	1947	1998	B	151	1998	2006	2014	881	RTV ATV	2009
	X13	1959	2000	B	193	2000	2000	2014	547	TDF FTC EFV	2011
	X14	1972	2000	NA	146	2000	2002	2014	625	TDF FTC NVP	2009
	X15	1967	2006	NA	289	2006	2006	2014	1,175	EVP	2011
	X16	1966	2007	NA	371	2010	2011	2014	739	TRU RTV ATV	2011
	X17	1968	2008	NA	192	2008	2008	2014	354	ATR	2010
	X18	1972	1996	NA	373	1997	2009	2014	1,015	ATR	2010
	X19	1980	2006	NA	230	2006	2006	2014	623	KVX NVP	2012
HLA DR^- $CD25^-$ $CD69^-$ $CD4^+$ T cells	X20	1948	2009	NA	354	2009	2009	2014	817	KVX NVP	2013
	X21	1955	2000	NA	157	2000	2000	2014	579	TRU ATV	2009
	X22	1978	1996	NA	0	1996	2010	2014	493	TRU KLT	2009
	X23	1967	1992	NA	362	1994	2000	2014	845	TRU NVP	2009
	X24	1975	2004	B	292	2006	2006	2014	561	ATR	2010
	X25	1959	2009	B	629	2009	2009	2014	994	TDF FTC EFV	2010
	X26	1970	1996	B	331	1998	2010	2014	1,079	KVX RTV ATV	2010
	X27	1976	2004	NA	288	2007	2007	2014	819	DRV RTR	2014
	X28	1945	1988	B	9	1997	2002	2014	491	RTV DRV RLT	2011
	X29	1965	2001	NA	182	2002	2002	2014	789	ATR	2012
	X30	1969	2011	F2	364	2012	2012	2014	723	KVX RTV ATV	2012
	X31	1945	2000	NA	141	2000	2000	2014	469	ATR	2010
	X32	1970	2005	B	291	2005	2012	2014	493	TRU RTV ATV	2011
	X33	1963	2005	NA	237	2005	2008	2014	414	ATR	2009
	X34	1970	2001	Not-B	264	2002	2007	2014	697	KVX RLT	2012

NA = Non applicable
