# Supplementary material for: Sequential treatment with 5‐aza‐2′‐deoxycytidine and deacetylase inhibitors reactivates HIV‐1
Source: EMBO Mol Med. 2015 Dec 17;8(2):117–38. doi: 10.15252/emmm.201505557 (PMC4734845; doi:10.15252/emmm.201505557)
Supplement: Supplementary file 2 — Review Process File [file EMMM-8-117-s002.pdf]

Sequential treatment with 5-aza-2′-deoxycytidine and deacetylase inhibitors reactivates HIV-1

Sophie BOUCHAT, Nadège DELACOURT, Anna KULA, Gilles DARCIS, Benoit VAN DRIESSCHE, Francis CORAZZA, Jean-Stéphane GATOT, Adeline MELARD, Caroline VANHULLE, Kabamba KABEYA, Marion PARDONS, Véronique AVETTAND-FENOEL, Nathan CLUMECK, Stéphane DE WIT, Olivier ROHR, Christine ROUZIOUX, and Carine VAN LINT

Corresponding author: Van Lint Carine, Université Libre de Bruxelles

Review timeline:

Submission date:	19 June 2015
Accepted:	09 November 2015

Editor: Céline Carret

Transaction Report:

No Review Process File is available with this article, as the authors have chosen not to make the review process public in this case

© EMBO
